# Diversity of transposable elements and repeats in a 600 kb region of the fly *Calliphora vicina*

**DOI:** 10.1186/1759-8753-4-13

**Published:** 2013-04-03

**Authors:** Bárbara Negre, Pat Simpson

**Affiliations:** 1 Department of Zoology, University of Cambridge, Downing Street, Cambridge, CB2 3EJ, UK; 2 Current address: EMBL/CRG Systems Biology Research Unit, Centre for Genomic Regulation (CRG), and Universitat Pompeu Fabra (UPF), Dr Aiguader 88, Barcelona, 08003, Spain

**Keywords:** *Calliphora vicina*, Diptera, Helitrons, Horizontal transfer, Repeats, Retrotransposons, Transposable elements, Transposons

## Abstract

**Background:**

Transposable elements (TEs) are a very dynamic component of eukaryotic genomes with important implications (*e.g.*, in evolution) and applications (*e.g.*, as transgenic tools). They also represent a major challenge for the assembly and annotation of genomic sequences. However, they are still largely unknown in non-model species.

**Results:**

Here, we have annotated the repeats and transposable elements present in a 600 kb genomic region of the blowfly *Calliphora vicina* (Diptera: Calliphoridae) which contains most of the *achaete-scute* gene complex of this species. This is the largest genomic region to be sequenced and analyzed in higher flies outside the *Drosophila* genus. We find that the repeat content spans at least 24% of the sequence. It includes 318 insertions classified as 3 LTR retrotransposons, 21 LINEs, 14 cut-and-paste DNA transposons, 4 helitrons and 33 unclassified repeats.

**Conclusions:**

This is the most detailed description of TEs and repeats in the Calliphoridae to date. This contribution not only adds to our knowledge about TE evolution but will also help in the annotation of repeats on Dipteran whole genome sequences.

## Background

Transposable elements (TEs) are a common feature in eukaryotic genomes and constitute a major player in many of the processes that shape the genome and control gene expression [1,2]. TEs can occupy a significant but highly variable portion of the genome. For example, at least 46% of the initial sequence of the human genome was recognized as TEs, and this percentage is probably higher than 50% when other repeats are considered [3]. Amongst species of Diptera sequenced to date the repeat content of euchromatic regions varies from only 6% in *Drosophila melanogaster*[4] to 16% in *Anopheles gambiae*[5], 28% in *Culex quinquefasciatus*[6] and 47% in *Aedes aegypti*[7]. TEs and other repeats pose a big challenge for the assembly and annotation of genomic sequences. Although many programs have been developed for the detection of TEs, most are difficult to use and their performance has not been properly tested [8]. They mostly rely on similarity to annotated elements or on the detection of known structures. The availability of well-annotated elements is thus of great help for their automatic detection and annotation.

Detailed description of TEs is not only important for genome annotation but also essential for understanding genome structure, function and evolution. The presence of TEs can affect gene structure and gene expression in several ways: from local effects on the expression of adjacent genes, to global effects such as the generation of large chromosome rearrangements or transpositions [2,9]. TEs are also important contributors to evolutionary adaptation [10]. Furthermore they contain historical information about the genome, and can be used as a sort of paleontological record. They provide a tool with which to solve evolutionary relationships and classification of species [11-14]. Moreover, TEs have a direct application for transgenesis where they can be used as insertion vectors. Knowledge of the TE repertoire of a target species has important implications for vector choice, as it will influence the stability of the transgenes. These methods are not only valuable research tools but are also being developed for the control of pest species in the wild [15].

TEs are divided into two main classes according to their structure and mechanism of transposition [16]. Class I elements, also called retrotransposons, transpose by reverse transcription of an RNA intermediate (DNA-RNA-DNA) mediated by a retrotranscriptase, whereas Class II elements transpose directly from DNA to DNA. Within each of these classes, TEs are further subdivided mainly on the basis of the structural features of their sequences [17,18]. Class I elements are divided into two main types: with or without Long Terminal Repeats (LTR elements and non-LTR elements), such as LINEs and SINEs. Class II elements include cut-and-paste DNA transposons, rolling-circle DNA transposons (Helitrons) and self-synthesizing DNA transposons (Polintons). Cut-and-paste DNA transposons are characterized by the presence of Terminal Inverted Repeats (TIRs) flanking a transposase that catalyses the transposition reaction. Helitrons have been classified as Class II-DNA transposons that use a “rolling circle” (RC) mode of transposition [19].

The Calliphoridae is a monophyletic family of calyptrate Muscomorpha (Diptera). These flies are of economic importance as a cause of myiasis in humans and animals, and as vectors of pathogens causing dysentery and other diseases. The larvae of most species are scavengers of carrion and dung, and fulfil an important ecological function in the decomposition of animal remains. They are among the first colonizers of cadavers, making them particularly useful for forensic entomology, predominantly to establish a minimum time since death, or minimum post-mortem interval [20]. This method usually relies on morphological identification of samples collected on corpses. Distinguishing between closely related taxa, such as *Calliphora vicina* and *Calliphora vomitoria*, can be a difficult process with major implications for post-mortem interval estimation. Mitochondrial sequences, like COI and COII, have been used for species identification but in some cases an overlap between intra- and inter-specific variability renders this method unreliable [20]. Measures to develop a TE-based simple and efficient marker system for the identification of forensically important carrion flies are currently being developed [21]. However, the retrotransposon landscape of carrion fly genomes remains largely unknown.

Here we provide an inventory and classification of the TEs and other repeats found in 6 BAC clones covering most of the Achaete-Scute Complex of *C. vicina*. These sequences include the genes *achaete* (*ac*), *scute* (*sc*) and *lethal of scute* (*l’sc*) which are highly regulated and surrounded by large regulatory regions. It is a 600 kb euchromatic region of the 750 Mb *C. vicina* genome. We have identified 318 insertions classified as 75 different repeats; 42 of which are TEs and 33 are unclassified repeats. Elements which are complete or present at high copy number are described in some detail. We also discuss probable cases of horizontal transfer.

## Results

**Figure 1:**
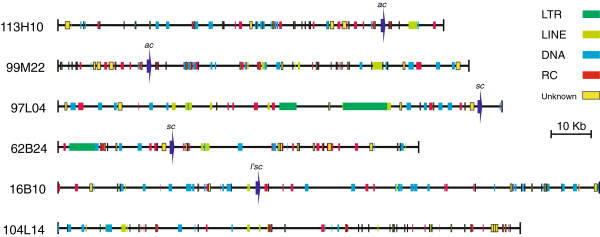
**Distribution of transposable elements and repeats in sequenced BACs.** TEs and repeats are represented as rectangles: *Class I-LTR elements* are shown in dark green, *Class I-LINEs* in light green, *Class II-cut and paste DNA-transposons* in blue, *Class II-rolling circle transposons* in red and *unclassified* repeats in yellow; dark blue arrows represent the *C. vicina* genes found in this region: *achaete* (*ac*), *scute* (*sc*) and *lethal of scute* (*l'sc*).

We have analysed a 613,063 bp genomic region within which we have identified a total of 318 TE insertions and repeats (Table 1, Table 2, Figure 1, Additional file 1, Additional file 2). The repeats have been classified and are described below.

**Table 1:** **Transposable elements and other repeats identified in *C. vicina***

	**Family**	**Name**	**Copies**	**Total size**	**Average**	**Longest**
**Class I (retrotransposons)**	LTR/Gypsy-CsRn1	CsRn1_Cv1	1	4294	4294	4294
	LTR/Gypsy-Osvaldo	Cv_Isis-like	1	10995	10995	10995
	LTR/Pao	Pao_Cv1	1	6420	6420	6420
	**Total LTR**	**3**	**3**	**21709**	**7236**	**10995**
				**3.54%**		
	LINE/CR1	CR1-1_CV	3	2703	901	1461
	LINE/CR1	CR1-2_CV	2	309	155	309
	LINE/CR1	CR1-3_CV	1	188	188	188
	LINE/Jockey	Jockey_Cv1	1	553	553	553
	LINE/Jockey	Jockey_Cv2	1	180	180	180
	LINE/LOA	LOA-1_Cv	1	536	536	536
	LINE/LOA	LOA-2_Cv	1	1636	1636	1636
	LINE/LOA	LOA-3_Cv	1	157	157	157
	LINE/LOA	LOA-4_Cv	1	294	294	294
	LINE/LOA	LOA-5_Cv	1	447	447	447
	LINE/LOA	LOA-6_Cv	3	2414	805	1157
	LINE/LOA	LOA-7_Cv	1	472	472	472
	LINE/LOA	LOA-8_Cv	1	129	129	129
	LINE/LOA	LOA-9_Cv	1	83	83	83
	LINE/LOA	LOA-10_Cv	2	890	445	660
	LINE/L2	L2-1_Cv	1	315	315	315
	LINE/RTE	RTE-1_Cv	1	1111	1111	1111
	LINE/RTE	RTE-2_Cv	1	726	726	726
	LINE	LINE1_Cv	1	276	276	276
	LINE	LINE2_Cv	3	2541	847	2125
	LINE	LINE3_Cv	1	2116	2116	2116
	**Total LINE**	**21**	**29**	**18076**	**623**	**2125**
				**2.95%**		
**Class II (DNA transposons)**	DNA/ITm-mariner	Cv-mar1	40	30549	764	1296
	DNA/ITm-mariner	Cv-mar2	14	6154	440	989
	DNA/ITm-mariner	Cv-mar3	5	950	190	311
	DNA/ITm-mariner	Cv-mar5	1	427	427	427
	DNA/ITm-DD37E	DD37E_Cv1	4	3073	768	1304
	DNA/ITm-Tc1	AMARI_Cv1	5	1863	373	657
	DNA/ITm-Tc1	SMAR_CV1	1	357	357	357
	DNA/ITm-Tc1	CRMAR_CV1	1	305	305	305
	DNA/ITm-Tc3	Tc3_CV1	1	356	356	356
	DNA/ITm-Tc3	Tc3_CV2	1	414	414	414
	DNA/MITE	MITE_Cv1	8	1524	191	245
	DNA/Chapaev-Chapaev3	Chapaev3-1_CV	2	1658	829	1075
	DNA/Chapaev-Chapaev3	Chapaev3-2_CV	1	302	302	302
	DNA/hAT	hAT_CV1	2	265	133	163
	**Total DNA**	**14**	**86**	**48197**	**560**	**1304**
				**7.86%**		
	RC/Helitron	Helitron1-Cv	3	1431	477	1269
	RC/Helitron	Helitron2-Cv	41	19310	471	767
	RC/Helitron	Helitron3-Cv	40	9901	248	821
	RC/Helitron	Helitron4-Cv	1	85	85	85
	**Total RC**	**4**	**85**	**30727**	**361**	**1269**
				**5.01%**		
**Unclassified**	Unknown	unknown1	4	2637	659	1010
	Unknown	unknown2	5	542	108	127
	Unknown	unknown3	4	1459	365	521
	Unknown	unknown4	1	535	535	535
	Unknown	unknown5	20	4144	207	269
	Unknown	unknown6	12	3283	273	482
	Unknown	unknown7	4	3088	772	1009
	Unknown	unknown8	6	2051	342	600
	Unknown	unknown9	1	160	160	160
	Unknown	unknown10	5	598	120	130
	Unknown	unknown12	2	228	114	228
	Unknown	unknown13	7	3069	438	570
	Unknown	unknown14	2	246	123	129
	Unknown	unknown15	2	212	106	127
	Unknown	unknown16	1	151	151	151
	Unknown	unknown17	2	173	87	115
	Unknown	unknown18	3	658	219	247
	Unknown	unknown19	1	60	60	60
	Unknown	unknown20	10	1057	106	138
	Unknown	unknown21	1	55	55	55
	Unknown	unknown22	1	146	146	146
	Unknown	unknown23	8	3012	377	902
	Unknown	unknown24	1	138	138	138
	Unknown	unknown25	1	182	182	182
	Unknown	unknown26	1	242	242	242
	Unknown	unknown27	1	82	82	82
	Unknown	unknown28	1	137	137	137
	Unknown	unknown29	1	105	105	105
	Unknown	unknown30	1	108	108	108
	Unknown	unknown31	1	103	103	103
	Unknown	unknown32	1	110	110	110
	Unknown	unknown33	1	1429	1429	1429
	Unknown	unknown34	8	1913	239	359
	**Total unknown**	**33**	**120**	**32113**	**268**	**1429**
				**5.24%**		
	**Total repeats**	**75**	**323**	**150822**		
				**24.60%**		

**Table 2:** **Total repeat content in *C. vicina *BAC sequences**

		**Repeats**		
**BAC**	**Length**	**Number**	**Total bp**	**% of sequence**
113H10	96426	61	23570	24.44
99 M22	102758	78	28879	28.10
97 L04	111044	38	38432	34.61
62B24	90178	44	28013	31.06
16B10	135393	66	27144	20.05
104 L14	115595	68	19608	16.96
**Total BACs**	**651394**	**355**	**165646**	**25.43**
Total region*	**613063**	**317**	**149226**	**24.34**

(*without repeated alleles).

### Class I – RNA-mediated TEs

#### LTR retroposons

LTR elements are characterized by the presence of direct long terminal repeats (LTRs) that range from a few hundred base pairs to more than five kilobases long [17]. Between the LTRs there are generally only one or two open reading frames (ORFs) that encode a polymerase (pol) protein and a protein related to the retroviral group-associated antigen (gag) protein. The *pol* protein contains reverse transcriptase (RT), ribonuclease H (RNaseH), protease (PR) and integrase (IN) domains that are important for the process of retrotransposition. The *gag* protein binds nucleic acids or forms a nucleocapside shell. Some LTR retrotransposons also have an env (envelope)-like domain that encodes a transmembrane receptor-binding protein that allows the transmission of retroviruses.

We have identified three LTR retrotransposon elements, each with one insertion. These elements are recent insertions; all three are full length, have identical or almost identical LTRs and at least two of the three insertions are polymorphic (see below).

##### Isis-like

**Figure 2:**
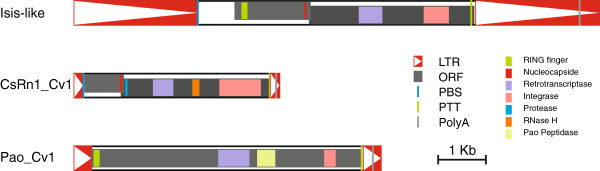
**Structure of the LTR elements.** Diagram showing the structural features of the three LTR elements identified in this study. All features are drawn to scale (except PBS and PPT). See legend for colour code. Full sequences of these elements can be found in Additional file 3: Figure S1, Additional file 4: Figure S2, and Additional file 5: Figure S3, respectively.

This is the largest identified repeat with 10,995 bp (Figure 2, Additional file 3: Figure S1). It is closely related to the Isis TE recently described in *Drosophila buzzatii*[22]. It belongs to the Osvaldo lineage of the Gypsy family. The LTRs of Isis-like are 2577 and 2574 bp long and there are 4 bp Target Site Duplications (TSD: CGTG) and two ORFs. The first ORF encodes a 531-amino acid (aa) gag protein with a 40% identity (and 70% similarity) with Isis. It contains a RING finger domain which is absent in Isis but present in Osvaldo (also from the same family). The second ORF encodes a 1,137-aa pol protein, which has 60% identity (and 85% similarity) with the Isis pol protein. However, Isis-like lacks the env domain and the LTR of both elements are very different (742 *vs.* 2574 bp long). This is a recent insertion, less than 25,000 years old, and is polymorphic as it is present in only one of the two sequenced alleles covering this region.

##### CsRn1_Cv1

This element is 4294 bp long. It comprises 179 and 180 bp LTRs and two ORFs 267 and 1,036-aa long (Figure 2, Additional file 4: Figure S2). It belongs to the CsRn1 lineage of the Gypsy family [23]. This lineage is characterized by the presence of a PBS complementary to tRNA-Trp, a CHCC gag motif and the GPY motif in the 3^′^ of the Integrase protein, all of which are present in this element. However, it seems to present a 6 bp TSD (CAAGTG) instead of the 4 bp TSD typical of the group. We have estimated this insertion to be 350,000 years old, which makes it the oldest of the three LTR elements.

##### Pao_Cv1

The last LTR element identified belongs to the Pao family, and is related to the Ninja-I element. Pao_Cv1 is 6420 bp long, has 355 bp long LTRs, and one ORF coding for a 1881 aa protein (Figure 2, Additional file 5: Figure S3). It has 5 bp TSDs (GCGGG). It is inserted inside a mariner element. This insertion is polymorphic and furthermore the two LTRs are completely identical which indicates that it is very young (less than 88,000 years old).

#### Non-LTR retroposons (LINEs)

A total of 29 insertions have been classified as 21 different LINE elements, most of which are short and degraded fragments. The insertions average 745 bp in size and ten of them are smaller than 500 bp, whereas size typically ranges from 1 to 7 kb for this group [24]. The absence of canonical sequences for comparison makes it difficult to classify them properly. This is particularly acute for the LAO elements, from which we have found many very short fragments (for eight out of ten putative elements the longest fragment is smaller than 1 kb, the smallest being 83 bp only) (Table 1). We cannot exclude the possibility that some of the insertions we have defined as separate elements are in reality different regions of the same element. The size and degraded nature of these elements suggests they are all old insertions. Overall the identified LINEs span 18 kb of the sequenced region (2.9%).

### Class II – DNA transposons

#### Cut-and-paste DNA transposons

Cut-and-paste DNA transposons are characterized by 10 to 200 bp terminal inverted repeats (TIRs) flanking one or more ORFs encoding a transposase. We have identified 14 different cut-and-paste DNA elements with a total of 89 insertions spanning 7.86% of the sequenced region. One element belongs to the MITE family, two to the Chapaenov family, one to the hAT family, and the remaining 10 to the IS630-Tc1-mariner (ITm) superfamily. The most common elements belong to the Mariner family of the ITm superfamily.

##### Cv-mar1

**Figure 3:**
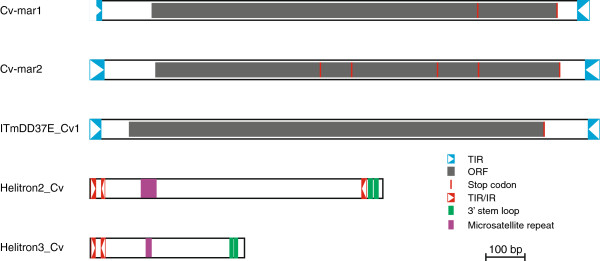
**Structure of the DNA-transposons.** Diagram showing the structural features of the cut-and-paste and rolling circle transposons for which we obtained consensus sequences. All features are drawn to scale. See legend for colour code. Full sequences of these elements can be found in Additional file 6: Figure S4, Additional file 9: Figure S7, Additional file 12: Figure S10, Additional file 13: Figure S11, and Additional file 14: Figure S12.

The most frequent transposon is *Cv-mar1* with 41 different insertions that span overall more than 30 kb. All insertions are partially degraded and range from 320 to 1296 bp, the consensus sequence is 1,275 bp long (Figure 3, Additional file 6: Figure S4). This element shows 78% identity at the nucleotide level with the Desmar1 mariner element from the Hessian fly *Mayetiola destructor*[25-27] (Additional file 7: Figure S5). Its TIRs have been identified by similarity to those of Desmar1 [25], with which they show 3 nucleotide (nt) substitutions and 1 nt insertion. However, the 5^′^TIR of Cv-mar1 is incomplete and the 3^′^TIR is present in only a single copy of the element (the fragment of the consensus sequence derived from a single element is delimited by a blue dash in Additional file 6: Figure S4). Although none of the annotated elements displays a complete transposase, we were able to derive a “complete” copy from the consensus sequence. In position 993 (shown in red) the consensus sequence has a T that results in a stop codon in the transposase, however a third of the sequences have an A at this position, which would result in an arginine (R) residue. The next stop codon is in the same position as that of the Desmar1 element (Additional file 8: Figure S6). If we consider this longer transposase it is 345 aa long.

##### Cv-mar2

In the region analysed there are 14 copies of *Cv-mar2* which span a total of 6 kb. The average insertion is 440 bp long, with the longest being 989 bp. Although none of the insertions is full length we were able to derive a consensus full length sequence which is 1299 bp long (Figure 3, Additional file 9: Figure S7), individual copies are 77% to 91% identical to the consensus. It has 35 bp TIRs and a 344 aa transposase. However, this consensus element would be non-functional as the TIRs have five mismatches and the transposase has four stop codons and commences with a leucine instead of a methionine. This element is very similar to the Mariner1_DYa from *Drosophila yakuba*[28]. The consensus obtained has a 78% identity at the nucleotide level with Mariner1_DYa and the two transposases show 73% identity at the amino acid level (Additional file 10: Figure S8 and Additional file 11: Figure S9).

##### DD37E_Cv1

The DD37E_Cv1 element belongs to the ITm-DD37E family [26]. This family was first discovered in mosquitos and is characterized by a unique DD37E catalytic domain. The full-length copy of this element is 1298 bp long with a 354 aa ORF and 27 bp ITRs (Figure 3, Additional file 12: Figure S10). At both ends of the insertion we find the TA sequence, the canonical dinucleotide target site duplication of the family [29]. Three additional copies are fragmented, highly degraded and in two cases enclose other nested repeats. This element has been present in the *C. vicina* genome for a long time (presence of degraded insertions). The identification of a full-length copy suggests this element has also been active recently in *Calliphora*.

#### Rolling circle (RC) transposons - Helitrons

Helitrons have been classified as class II-DNA transposons that use a “rolling circle” mode of transposition [19]. They encode proteins similar to helicases, ssDNA-binding proteins and replication initiation proteins [4,19]. Helitrons lack inverted repeats but are characterized by much-conserved termini and hairpin structures close to the 3^′^ end. As with other TEs, the Helitrons present both autonomous and non-autonomous elements. *DINE-1* and *mini-me* elements from *Drosophila*, which show some unique characteristics, are now classified as non-autonomous Helitrons [30,31]. They lack coding capacity, do not have these characteristic termini, but have subterminal inverted repeats and the hairpin structures at the 3^′^ region [30]. Four different elements of the Helitron family are present in our sample. Two of them show a high copy number, with 40 and 41 insertions, respectively. Helitrons cover 5.01% of the analysed sequence.

##### Helitron2_Cv

Was identified by similarity to the 5^′^region of the Arylphorin subunit from *C. vicina* (X63340). RepeatMasker indicated it is related to *Helitron-1N1_Dvir* and *mini-me* elements [32]. We have annotated 41 copies of this element, from 136 to 767 bp long. The consensus sequence is 750 bp long (Figure 3, Additional file 13: Figure S11). Eight copies are full length and show a 95% to 97% identity with the consensus. *Helitron2_Cv* shows the structural features of non-autonomous *DINE1-like* Helitrons: 11 bp subTIRs, partial inverted repeats next to the 5^′^ subTIRs, GTCY-rich protosatellites and short hairpin stem-loops (with 9 bp stems) next to the 3^′^end of the element. It is closely related to the autonomous and non-autonomous elements *Helitron-1-Dvir* and *Helitron-1N1_Dvir* of *D. virilis*[32]. *Helitron2_Cv* shows a 65% and 70% identity in the 5^′^region (up to protosatellite repeat) and 3^′^end (last 100 bp), respectively, with the *D. virilis* elements. Copies of this element represent 3% of the sequenced region. Given the level of divergence of the full length insertion, autonomous copies of this element probably exist in the *C. vicina* genome.

##### Helitron3_Cv

This is also a *DINE1-like* Helitron. We have identified 40 copies that range from 71 to 821 bp. They can be divided into two subtypes, whose consensus sequences are 395 and 396 bp long. The consensus of the two subtypes differs in one nucleotide indel and 54 nucleotide substitutions, half of which are located in the region just after the protosatellite repeat. All features typical of *DINE1-like* Helitrons are present except the 3^′^ subTIR (Figure 3, Additional file 14: Figure S12). The protosatellite repeat (GTCT)_2_ is expanded in 3 of the insertions: one has 4 repeats, another 5 repeats and the third 108 repeats.

### Unclassified repeats

These repeats have been mainly identified by similarity within and between BAC sequences and with other published *Calliphora* sequences (*blastn* – non-redundant nucleotide NCBI database). They are mostly short and with no obvious structure or similarity with known elements. Overall these repeats span 5.24% of the analysed region.

#### Unknown 5

This repeat was first identified by *blastn* to the non-redundant NCBI database, as it is present in intergenic or intronic regions of two different alleles of the Xdh gene of *C. vicina* (M30316, M30488). We have annotated 20 insertions of this element in the region we analysed. The consensus sequence is 275 bp long (Additional file 15: Figure S13). The 5^′^region of the element is rich in polyA and polyT tracts, whereas the 3^′^region of the element is highly conserved between copies (red region in Additional file 15: Figure S13). However, no structural features or internal repeats could be recognized.

#### Unknown 6

A short fragment of this element was first identified by RepeatMasker as a fragment of a Helitron. However, in this sequence, which is present 12 times in the *C. vicina* sequences, we could not identify any of the features of a Helitron and thus it remains unclassified. The consensus sequence of this element is 488 bp long (Additional file 16: Figure S14). From nucleotide 1 to 465 the sequence is palindromic (with 92% identity).

#### Unknown 20

This element was first identified by *blastn* with similarity to a *Lucilia cuprina* intronic sequence (M89990). There are 10 insertions of this sequence present in the region of *C. vicina* that was analysed. The consensus sequence is 140 bp long (Additional file 17: Figure S15). No structural features or internal repeats were identified which could help classify this repeat.

### Candidates of horizontal transfer

Four of the analysed repeats show a remarkable similarity with elements from other species. To assess the possibility of horizontal transfer we have taken a closer look at these elements and checked their distribution on available sequences (NCBI and Insect genome sequences – see Methods). These elements are the LTR element *Isis*, the DNA cut-and-paste elements *Cv-mar1* and *Cv-mar2*, and the Helitron *Helitron2_Cv*.

The elements *Isis* from *D. buzzatii* and *Isis-like* from *C. vicina* have 40% and 60% identity in their ORFs, however they differ in the presence of the RING (present only in *Isis-like*) and env (present only in *Isis*) domains. The sequence (and length) of their LTRs is also very different. Of the sequenced genomes, only *D. mojavensis* presents an *Isis* element. We have found no evidence of *Isis-like*. The limited distribution of these elements suggests that they arrived by horizontal transfer to the *D. buzzatii-D. mojavensis* ancestor (after the split of *D. virilis*) and to *C. vicina* (or its ancestors).

The *Cv-mar1* element shows 70% to 80% identity with multiple Mariner elements described in different insect species [33-35] besides Desmar1 [25]. The whole genome sequences of *Mayetiola, Rhodnius prolixus* (Hemiptera), *Solenopsis invicta* (Hymenoptera) and *Anopheles gambiae* (Nematocera) include fragments of this element (500 to 800 bp long) with 80% identity. The broad distribution of this element suggests it is mainly vertically transmitted.

The Mariner element *Cv-mar2* is present in *D. yakuba* (*Mariner1_Ya*) with which it shows 78% identity over its whole length. We have also found several hits with 80% identity in the ants *Camponeatus floridanus* and *Harpegnathos saltator* (Hymenoptera), covering 80% and 60% of the length of the element, respectively. We found no evidence of this element in other species. Its high similarity and limited distribution suggest its transmission by horizontal transfer between Diptera and Hymenoptera which diverged approx. 300 Myr ago.

The *Helitron2_Cv* is similar to *Helitron-1N1_Dvir* from *D. virilis*. They have 50% identity over the whole element, and 65% to 70% identity at the 5^′^ and 3^′^end, respectively. Multiple hits with 60% to 90% identity around sequenced genes of Lucilia, Musca and other species show that this element is very common within the Muscomorpha. No hits were found in the whole genome sequences with *Helitron2_Cv*. Using *Helitron-1N1_Dvir* as query, we find multiple hits in *Drosophila* species but nothing outside the *Drosophila* genus. This suggests that this element is vertically transmitted, the absence of hits in other insect is probably due to evolution of the sequence of this element.

## Discussion

We have analysed a small (600 kb) region of the *Calliphora* genome. It contains most of the Achaete-Scute complex: with the genes *ac*, *sc* and *l’sc*. The low gene density in this region is due to the presence of large regulatory regions (Negre and Simpson, submitted). It is euchromatic in nature although we do not know its position in the chromosome or whether it is representative of the genome in terms of TE content and diversity but there are no reasons that would indicate otherwise. The discussion that follows is only a first approximation to the repeat landscape of this fly species, *C. vicina*, which has a big genome with 750 Mb (Spencer Johnston personal communication).

### Fraction of genomic DNA occupied by repeats

Repeats span 24% of the region analysed (600 kb). This percentage is relatively high but not unusual for fly genomes. Larger genomes usually show a higher proportion of repeats; however, repeat content is not proportional to genome size and is highly variable between dipteran genomes (Table 3). For example, there are several species whose genome is around 200 Mb with a repeat content ranging from 3% to 25%.

**Table 3:** Repeat content in dipteran genomes

		**% Genome**	**Class I**		**Class II**			
**Species**	**Genome size**	**All TEs**	**LTR**	**Non-LTR (LINE)**	**DNA (TIR)**	**Helitron**	**Other**	**Unclassified**
*Drosophila melanogaster*	180 Mb	6%^1^	4.20%^1^	1.38%^1^	0.30%^1^	-	0.12%^1^	-
*Drosophila ananassae*	231 Mb	25%^1^	15.5%^1^	7.00%^1^	1.25%^1^	-	1.25%^1^	-
*Drosophila virilis*	206 Mb	14%^1^	9.94%^1^	3.36%^1^	0.28%^1^	-	0.42%^1^	-
*Drosophila grimshawii*	200 Mb	3%^1^	1.53%^1^	0.66%^1^	0.57%^1^	-	0.24%^1^	-
*Calliphora vicina* (600 kb region)	750 Mb	24%^*^	3.54%	2.95%	7.86%	5.01%	-	5.24%
*Anopheles gambiae*	278 Mb	16%^2^	2.64%^4^	3.75%^4^	4.54%^4^	0.11%^4^	-	-
*Aedes aegypty*	1,376 Mb	47%^3^	12.41%^4^	12.67%^4^	13.97%^4^	1.26%^4^	-	-
*Culex quinquefasciatus*	579 Mb	28%^4^	3.89%^4^	4.45%^4^	19.40%^4^	0.49%^4^	-	-

^*^% of TEs based on the 600 kb region analysed in this study, data from other species comes from whole genome sequences. References: ^1^Drosophila 12 genomes consortium 2007; [37]^2^Holt, *et al.*[5]; ^3^Nene, *et al.*[7]; ^4^Arensburger, *et al.*[6].

Repeat content is also variable within genomes, being most abundant in heterochromatin and pericentromeric regions. Unfortunately, we have no information about the position within the chromosome of the region we analysed. In *D. melanogaster* it is close to the tip of the X chromosome, however chromosomes are very dynamic in terms of gene order, so we do not expect the position to be necessarily conserved.

### Abundance of the different classes of repeats

If we look at the distribution of repeats in Dipterans, the abundance of the different classes appears to be constant within lineages independently of total repeat content, but very divergent between lineages (Table 3). In *D. melanogaster* LTRs are the most abundant TEs, followed by non-LTR and then TIR elements [36] (there is no information about Helitrons). The same pattern is observed in the other 11 *Drosophila* species that have been sequenced [37]. The pattern changes in mosquitos where TIR elements are the most abundant, followed by non-LTR, LTRs and finally Helitrons with less than 1% (Table 3). As in Drosophilidae, all mosquitos show the same pattern, although in *Anopheles* and *Aedes* the quantity of TIR, non-LTR and LTR elements is very similar, whereas in *Culex* TIR elements represent more than half of the repeat content. In *Calliphora* we see again a completely different pattern. As in mosquitoes TIR elements are the most frequent but they are now followed by Helitrons. LTR and non-LTR elements (in this order) are the least frequent in *C. vicina* (Table 3). It is noteworthy that if we consider the unclassified repeats in *Calliphora* this would be the second most frequent class of repeats.

### Age of TE insertions

#### Nested elements

Of the 322 identified repeats 11 (3.4%) are nested within other elements. Two of the three LTR elements are nested within other repeats, whereas none of the LTR elements themselves show insertions of other elements. This is consistent with the fact that they are recent insertions. At the other extreme, the unclassified (unknown) elements, in spite of being the most numerous (37%), show the smallest proportion of nested elements: only one copy is nested and two include insertions of other elements. The fact that one copy of unknown 20 is nested within another TE suggests that this element is mobile although no structural features have been identified (see results). On the other hand, the fact that only one of the 119 unknown repeats is nested suggests that some of them might not be mobile. For the other types of elements (LINE, DNA and RC) the frequency of nested copies is proportional to the number of insertions. However, LINEs show a high number of copies serving as landing sites. This, together with the small size and degraded nature of most copies, indicates that most LINE insertions are very old. Of the RC elements, all three nested insertions belong to Helitron2, two of which are full length. Two of the three are nested inside fragmented copies of the DNA element DDE37E_Cv1.

#### New vs. old insertions

All LTR insertions found in this sample are recent in origin. All three insertions are full length and at least two of them are polymorphic. We have found no fragments or degraded copies. This is a very different picture to that found in all other TE classes where none (non-LTR elements) or only a few (DNA and RC elements) insertions are full-length. In all these classes most insertions are fragmented and highly degraded. A similar trend was found in *D. melanogaster*. LTR families appear to be transposing in the *D. melanogaster* genome at higher rates than TEs from other orders leading to the observation that LTR elements, as a group, tend to be younger [38]. Recent analyses suggest that this trend is due to a higher intrinsic rate of transposition of LTR elements and not to a recent increase of transposition [39].

### Role of horizontal transfer

The mobile nature of TEs makes them prone to horizontal transfer. It is thought to be an essential step in TE life cycle, which allows them to escape vertical extinction [40,41].

Four TEs showed a remarkable similarity with elements from other species. Although we could not compare the rates of synonymous mutations between the TEs and orthologous genes, we have checked the distribution of these elements in sequenced insect species to detect possible instances of horizontal transfer.

The broad distribution of the Mariner element *Cv-mar1* and the Helitron *Helitron2_Cv* shows they are vertically transmitted. We cannot rule out completely horizontal transfer in *Cv-mar1*, but its detection would require a much thorough analysis (which is out of the scope of this study).

The elements *Isis* and *Cv-mar2* do seem to have undergone horizontal transfer. *Isis* moved between Calliphoridae and Drosophilidae which diverged approximately 100 Myr ago, and *Cv-mar2* between Diptera and Hymenoptera which diverged approximately 300 Myr ago.

Overall two of the 43 identified TEs show evidence of horizontal transfer. One is an LTR and the other a DNA transposon, the two classes more often involved in transfer events [40].

## Conclusions

This is the first detailed description of TEs in carrion flies. Although the analysis includes only a small region of the genome it gives an overview of the classes of TEs present and their abundance. Moreover, the description of these TEs and repeats can help in the annotation of repeat sequences in other Dipteran genomes, *e.g.,* those currently being sequenced.

## Methods

### Sequences analysed

We have analysed the sequences of six overlapping BAC clones, in a region which contains most of the Achaete-Scute Complex (AS-C) of *Calliphora vicina* (cloning and sequencing of this region is described in Negre and Simpson, submitted). The clones comprise a total of 651,394 base pairs (bp), of which 38,331 bp correspond to identical alleles in two overlapping clones (see Table 2). Thus we have analysed 613,063 bp of unique sequence.

### Identification of repetitive elements

Several tools were used for the identification and classification of repeats: RepeatMasker was run against the *Drosophila* database and all hits were considered, for protein-based RepeatMasker (A.F.A. Smit, R. Hubley and P. Green, RepeatMasker at http://repeatmasker.org) all hits were also considered; *blastn* and *blastp* were run against NCBI non-redundant databases [42] and hits longer than 100 bp with identities over 60% were further analysed. LTR-Finder [43] was used to identify LTR elements and some of their structural features such as PBS and PPT sequences. The online program Palindromes (http://mobyle.pasteur.fr) was used to aid in the identification of TIRs. All hits were compared between methods and manually inspected. Most repeats are identified by more than one method. Non-overlapping hits smaller than 50 bp were discarded. The best match was used for repeat classification. Annotated repeats were added to a local database to help in the identification of further copies of the same repeats. Comparison between *Calliphora* sequences (with *blast2sequences-blastn*) allowed the identification of many short unclassified repeats which are found recurrently in the *Calliphora* genome. Some of the elements we have annotated are also present in GeneBank sequences (in intronic and intergenic regions), but these were all unannotated. Consensus sequences were obtained by ClustalW [44,45] or Tcoffee [46,47] alignment and manually corrected with the aid of Bioedit.

### Divergence time of TE insertions

The age of TE insertions (t) has been calculated as in [4]; t = K/v, where K is the average divergence of TE copies from the consensus and v the neutral substitution rate. We have used the neutral substitution rate for *Drosophila* (v=0.016 substitutions/Myr) [48]. For LTR elements we have used t = K/2v, where K stands for the divergence between the two LTRs of one insertion [4].

### Identification of similar elements in other species

Distribution of similar elements in other species was assessed by similarity searches (*blastn*) against: (1) the non-redundant NCBI database and (2) insect whole genome sequences (flybase) [42,49]. Only hits with >60% identity over half the length of the query sequence were considered.

## Abbreviations

Env: Envelope; IN: Integrase; IR: Inverted repeat; ITm: IS360-Tc1-mariner superfamily; LTR: Long terminal repeat; ORF: Open reading frame; PBS: Primer binding site; PR: Protease; PPT: Polypurine tract; RC: Rolling circle; RNaseH: Ribonuclease H; RT: Reverse transcriptase; TE: Transposable element; TIRs: Terminal inverted repeats; TSD: Target site duplication

## Competing interests

The authors declare that they have no competing interests.

## Authors’ contributions

BN conceived and designed the study, performed the analysis and drafted the manuscript. PS conceived the study and helped to draft the manuscript. All authors read and approved the final manuscript.

## Supplementary Material

Additional file 1 Detailed inventory of identified repeats in each BAC sequence. Click here for file

Additional file 2 Sequences of annotated repeats in fasta. Click here for file

Additional file 3: Figure S1 *Cv_Isis-like*. Full nucleotide sequence of the Isis-like element of *C. vicina* and protein translation of the two ORFs. Nucleotides in red are LTRs, in bold and underlined PBS and PPT sequences. Amino acids: RING finger domain in green, Nucleocapside CCHC domain in red, retrotranscriptase in blue and Integrase in pink. Click here for file

Additional file 4: Figure S2 *CsRn1_Cv1*. Full nucleotide sequence of the *CsRn1_Cv1* element of *C. vicina* and protein translation of the two ORFs. Nucleotides in red are LTRs, in bold and underlined PBS and PPT sequences. *Amino acids*: Nucleocapside CCHC domain in red, protease in pink, retrotranscriptase in blue, RNase domain in green, and Integrase in pink. Click here for file

Additional file 5: Figure S3 *Pao_Cv1*. Full nucleotide sequence of the *Pao_Cv1* element of *C. vicina* and protein translation of its ORF. Nucleotides in red are LTRs, in bold and underlined PBS and PPT sequences. Amino acids: RING finger domain in light green, retrotranscriptase in blue, Pao peptidase in dark green and Integrase in pink. Click here for file

Additional file 6: Figure S4 *Cv-mar1* consensus sequence. Consensus sequence of the *Cv-mar1* element and amino acid sequence of its transposase. At position 993 (shown in red) the consensus sequence has a T that gives a stop codon in the transposase; a third of the sequences have an A at this position, which would result in an arginine (R) residue. Underlined nucleotides correspond to the TIRs; the 5^′^TIR is incomplete. The blue dash close to the end of the sequence delimits a fragment found in one insertion only (see text for details). Click here for file

Additional file 7: Figure S5 ClustalW2 alignment of Cv-mar1 and Desmar1. Click here for file

Additional file 8: Figure S6 ClustalW2 alignment of Cv-mar1 and Desmar1 transposases. Click here for file

Additional file 9: Figure S7 *Cv-mar2* consensus sequence. Consensus sequence of the *Cv-mar2* element and amino acid sequence of its putative transposase. Underlined nucleotides correspond to the inferred TIRs (35 bp long); there are 5 nucleotide changes between the two TIRs of the consensus sequence. Click here for file

Additional file 10: Figure S8 ClustalW2 alignment of Cv-mar2 and Mariner1_DYa. Click here for file

Additional file 11: Figure S9 ClustalW2 alignment of Cv-mar2 and Mariner1_DYa transposases. Click here for file

Additional file 12: Figure S10 ITmDD37E_Cv1. Full nucleotide sequence of the DD37E element of *C. vicina* and aminoacid sequence of its transposase. Underlined nucleotides are the TIRs, and bold and underlined amino acids the catalytic domain. Click here for file

Additional file 13: Figure S11 *Helitron2_Cv* consensus sequence. Consensus sequence of *Helitron2_Cv* showing the main structural features: 5^′^ and 3^′^ subTIRs and IR are underlined, 3^′^ stem loop in red and microsatellite repeat in blue. Click here for file

Additional file 14: Figure S12 *Helitron3_Cv* consensus sequence. Alignment of the consensus sequence of the two subtypes of *Helitron3_Cv* (3a and 3b). The main structural features are highlighted: 5^′^ subTIR and IR underlined, 3^′^ stem loop in red and microsatellite repeat in blue. This element lacks the 3^′^subTIR. Click here for file

Additional file 15: Figure S13 Unknown5 consensus sequence. Consensus sequence of the Unknown 5 elements. The highly conserved region of the element is shown in red. Click here for file

Additional file 16: Figure S14 Unknown6 consensus sequence. Consensus sequence of the Unknown 6 elements. The palindromic region is underlined. Click here for file

Additional file 17: Figure S15 Unknown 20 consensus sequence. Consensus sequence of the unknown 20 elements. No structural features were identified. Click here for file
