# Supplementary material for: Diversity of transposable elements and repeats in a 600 kb region of the fly Calliphora vicina
Source: Mob DNA. 2013 Apr 3;4:13. doi: 10.1186/1759-8753-4-13 (PMC3630058; doi:10.1186/1759-8753-4-13)
Supplement: Additional file 3: Figure S1 — Cv_Isis-like. Full nucleotide sequence of the Isis-like element of C. vicina and protein translation of the two ORFs. Nucleotides in red are LTRs, in bold and underlined PBS and PPT sequences. Amino acids: RING finger domain in green, Nucleocapside CCHC domain in red, retrotranscriptase in blue and Integrase in pink. [file 1759-8753-4-13-S3.doc]

TGTAATGGACGATTATTGTTAAAAAGTTTAATTCTTGAAAGTACATATTTTATATTGTGATACATATTTATATATATAAATATGTATATAAACAAATATG 100

**LTR**

TATATGTTTGGTTTTTATTCCACATTCAAATTAAAATTTTGCCTCAAATTCGGTTTTTTCGAAAATTTCGTTTTGAATTTTAACAGTAATGCTTTGTGTG 200

TTTTATTTCACCTCACACTGTTGCATTTACTTTAAAAAATCAAGTTAGCCACATTGAAAATAGCCACTTTGTTTTGACACATTGCAACGCATGAGAACGC 300

ATAGCAACGCATATTAACGCAACTAAAAAAAATAGAAAAATCCCGAACACTAACGAGAATTGAAGCATGGTTTTTCGTAACCAAATGTGTTAACCTTCTT 400

TTGATTTTATACCTCAAAGTGAACACATCGTTCAGTTAAAATATATAATATCCTTTGTACAGGACACACTTTATTTTTTCTACCAATTTAATTTTAAAGT 500

TTCATAAAGGTAAATCTCGCAGACTTAAAGATTTCGATGTAGTTAATTAACATACAATATTTTTTTAATACTTAGACAACTAGAGTGGAAATTACCATAT 600

TGCTAAATCCCAAGATTCAACATAACCTGAATCCTTCTTAATCCTTGAGGTTATTGGAAGACTAATAAATTGTGGAAAAACACCATTAAAAGCCTCGAAT 700

ATACAAGCTAATTTCCATCTAAAAAGGCACGGTGTGTTTATTTAGCCACCACTGTATCCACTTTTAAGTCACATAAAAAAAACCTAAAAACCACGCTCAC 800

AAGAATTGTGCTTACCGCTGATCATCATTATTTTAAATCTACCGATAAGACAACACCTAAAGTCAAGTGCTTCATTATTGTGTATCAGCCAAAAAGAAAT 900

TCGAAAAAAAAACTACAAATTTTATAAGCAAATATTAAGAGTCTCAGACGAGCTTCAAAACACCTACCAGCTTCCAAAATAGGACCGGATAAGTCGTCTA 1000

GGAAAACCTCCAAAAATCATCTAAGTTCTTCATAAGACAATTACCCAAATTTGAGTCTCAAAATAATTAAAATTTTACGTAAGATTGTGCATGTTTAAGT 1100

TTTTTTTTTGTGACTTTATATTCTTTTTTTCTTCTTAAAATCAAAAGGTGCAAACAATTTAAATTACCTTTAATAAATCAAACTACTTGAATTTCTTAAC 1200

AAAATTTATAAGTGTGATTAAAAACTTACAACTAAACCAATAAGAAAAATCACCTAACTTAAATCTAATTTAAATATATATAACAAATTAAAAACCAAAA 1300

GTTTAAAATGTATAAATGTAAAATATAAAGATTAAAAGCAAAAAAAAAGAAACTTAACCTATATTGCACATAAATTATCACAGTTGAATTATTTATTAAT 1400

TAAGTTCTTTTTATATATAATATAATTAATGACATGCAAAACAAATAAGATCTATGTAACAACAAAGTAAACATCCAACAAAAGCAGTCTGACGAGGCCT 1500

CATCATTCAGCGTTTCCAGAATTAAGTTGGTGAGTTTTTTTTCTCTGTTTTAAGTAATATTATTAATTAAAAAAATTTAGTTTTGCATTCATTATTATTA 1600

AATTTAATAACAAAATCAGAATATCTGTAATTTAAAAGAAACAATAATCAAATTTTTGAGGTTTGCTTTTACATATTCCTTGATTTGCTAGGGAATACTG 1700

TGTCAGTAACAAAGTCAAAAAAGAACAAGAAATAAAACACATTTCTTTGTTTTCTCACGCTTTTTTTTTTTGTTTATGCACAAGTAATTCATAAATTGAT 1800

ATGTAAAAGCAAAACACAAAAAATCGAAAACCATAAAACCATAAAACAAAAACAGTTTAAGCAATGATCATTTAACATTGTAAATGATCATTACAAGATA 1900

CCCATTGTATTCTCTAATTTGATTATTCTTTTTTTTTACTACAGTATTGTCCCAAAAATATAGAAATATAGTTTACTAGATTATTTCTTTAATATTATTA 2000

TAATTATCTATATTTAATTACTAGGCATTTCTTTTCTTAACAATTTACTTTTACTATTGTTGGTTAGAAAAAAAAAAATGTTATAATAATGTTTTTTCTA 2100

AATTTTGATCTTGTAAACCAAATTTATATTTAAAAGTCATTGAATTTTTTTTATTACCTACCTAATGGTTTTCCTCAATTTTGTTAAATAAATTGTTTAC 2200

TTGAATTATGAAATTCTAAGTTATTTTTAATGTTTTCGCGATGAGATGAAAAAGTAAGTGTGGATTTAATTAGGGATCTTAGTGTAATATTCGCCAATAA 2300

GATCATTGGATGGGAGGGTTTAGAGGCGATTATCCCAAACAACATCATGGGATAATAATAAAAGAGTTTTTCAATTAGTAAGTTCAGTCAGGGAGTCTAT 2400

AATAAATTATTTATGTATAATGATTCCTATATGAGAGAAAATTTAACATGAACAAGAATCCACCCTCCGACTGACCGCTAGTGGCAATTAATGGCTCCCG 2500

**PBS**

TCGTTCATTTAAATTTTAATCCAAAAGCTGTAACCTCATATAGCGTTGAATATGTATTTATTCAATTGTACATTACATAAATTG**GCGCCCAACGTGGGGC** 2600

CGAAAGAATTTAGACAAATTTTATAACAAACTTTTATAATATGTGTTCTTTATGAGCCATATTGATAACATCAATGTTTAATCTAAATTTTCTGCTCCAA 2700

AAGTTTTTCATTTAAATTTCATTTTTTTTTGAAAGTCTGAAATATAAGTCTTCTAAGTTTTCTGTTTGTAAATATTTCTTAGCACTTTTTAGGATTTTTC 2800

GACCATTTGGCATATATAGACCGAATAGTTGACCACTGAGTTAACTAAGGATTCTTTTGCTTAAGTAAATGCAGATTACTTTTATTTTTTTCTTTCAGTA 2900

GAATTTATACGATCGTATAAATAAAATTATCAGTTTGATGAATTACTAGTATCCTAATATTATTTGGTTTTACATGAATTTAGCTGAATGCAATTTCATG 3000

TCCGATACTATTAGTTTATTTTGACGTACAATAATAATGTATACTGGATTCCAAACTATATTTTTCATTATGCGGTTCTTATAAAATCTCCATAGCCCCA 3100

TTTGTTTAAAATAATTACACTTTACTAATGAAAACTCTCTATTACACACTTACTATCTCTCAAAAGCGAAAACTGTATTGAATCATTTGAAATATTTTTG 3200

TTTATACTAGTACATATAAAAAGTACATAAGTTTATTACTTAATAAAATCTGAAATTTTGTCCGAAAAATTTTGAATTTTAGTCTGTCTGATATAACGTT 3300

TTGGTAATACGATTTTAATCCCTATATACTTTTTTTTTATTAATTTACATTTCTTAACTCAAATCTAAAGAATTTAGAAAAAATGTCGTTATTGTATACG 3400

**ORF 1** M S L L Y T

CCACAAAAGGCACAGGAACAAGCTCAAAGTTCTGCAGCTGAACCTCCAGCTATTGACAATTCATGTGTAATTTGTAAAGAAACCATTGTAGATAACCAAG 3500

P Q K A Q E Q A Q S S A A E P P A I D N S **C**  V I **C** K E T I V D N Q E

**RING finger domain**

AAATTTTAATTATTACTACCTGTAGTCATGAGTTCCACCGAGCCTGTATAGAACATGCACTTTCTCAATCGGCTGAATGTCCTCAGTGTAAATGTGCTTG 3600

I L I I T T **C** S **H** E F **H** R A **C** I E H A L S Q S A E **C** P Q **C** K C A C

TGAGTTAGTAGACTTAATAGTTAAAAGATCCGATAACACACTTCCAAAAAATAGCCCTAAGCAAAAAACCGGTAACTCAAACCGCGGTAAACCTAGAGGA 3700

E L V D L I V K R S D N T L P K N S P K Q K T G N S N R G K P R G

GCAATGGCAAAGAAACATTTTACCAGGAATTATACGAAAAGTCTTGGTCAGGAATTTTCTCAACAGTCTTCTTTTGAACCCAATGGTTCTCATGTAGTCA 3800

A M A K K H F T R N Y T K S L G Q E F S Q Q S S F E P N G S H V V I

TACAAACAGATGAAAGAATTTATAGTCCACAAATGGATATTCGTTTTAATCGTCCAGAAAATTTTGTTGAACAATGTCCTCAGCCTAATAATAGTCAAAA 3900

Q T D E R I Y S P Q M D I R F N R P E N F V E Q C P Q P N N S Q N

TTATTCAAATGCTGTAGATTATTCTCAACTTAATCAAATGATAGAGAACACAGTTACTCGTTTATTAAGAAATCTCAATATTGTTCCAAATTCCATCAAC 4000

Y S N A V D Y S Q L N Q M I E N T V T R L L R N L N I V P N S I N

CAAAACCAAAATATTCAAGCTAATGTTAGACAAAGACAAACACCACCTGTTTCTTCCAATAATCAGTTTTTACAACCACCACAATATCACACTTCTGAAC 4100

Q N Q N I Q A N V R Q R Q T P P V S S N N Q F L Q P P Q Y H T S E P

CTTTACGCCAAAATTTGTCAGGAAATCATTATATTGATCCAAATTACAGTTTGAAAACGGATAAAATTACGGCAATAATACAAAACTGGAACATTAAGTT 4200

L R Q N L S G N H Y I D P N Y S L K T D K I T A I I Q N W N I K F

TGACGGTTCAAATAATGGCCTTACTGTGGAGGAATTTTTATATAGAGTTAGGTCTCTAACCACAGAAAACTTCAGTGGAGATTTTAATATAATTTGTAAA 4300

D G S N N G L T V E E F L Y R V R S L T T E N F S G D F N I I C K

CATTTACCCATGCTTTTGACCGGCAAAGCGCGTGACTGGTACTGGAGGTACCACAAACAAGTGGATAGGATTGAGTGGACCGAATTTTGCGCTGCATTGC 4400

H L P M L L T G K A R D W Y W R Y H K Q V D R I E W T E F C A A L R

GGTACCAATATAAAAATTTCAAGTCTAACTTTGATGTACGCGAAGAGGTTAGAAATCGAAAAATGAGATCCGGTGAAACGTTTGAAGTTTTTTACGATAA 4500

Y Q Y K N F K S N F D V R E E V R N R K M R S G E T F E V F Y D N

CATTTGTTCGATGCTTGACAGATTGGAAACTCCCATGCCCGAATCTGAGCTTGTAGAATTACTTACAAGGAACTTACGACCAGACATTAGGCATGAATTA 4600

I C S M L D R L E T P M P E S E L V E L L T R N L R P D I R H E L

TTATATGTCCCCATATATTCTATTGCTCACCTACGAAAATTGGTACAAATGAGGGAAAACCTCTTAGCCGACGACTATTTTAGACGCCATCCAACAACAA 4700

L Y V P I Y S I A H L R K L V Q M R E N L L A D D Y F R R H P T T K

AGCCAGCTGTACTTCCCATGCAAAGACGTACTGTAGCTGAGGTAGAGTTTTTAGAGGATAAACCTGAAGATATCACAAATTCCTATGAACTTTCAGTTGA 4800

P A V L P M Q R R T V A E V E F L E D K P E D I T N S Y E L S V D

CGCAATTCGTCAGGGACCTAATGCAGTAAAATGCTGGAATTGCGACGAACCAGGCCATCATTGGGAAGATTGTGTCAAAGATCGCATTGTTTTCTGCTAT 4900

A I R Q G P N A V K **C** W N **C** D E P G **H** H W E D **C** V K D R I V F C Y

**Nucleocapside: CCHC domain**

GGGTGTGGGACTAAAAACGTCTATAAACCTCAATGTCCTCGTTGCTTAGCTAGAAAAATATCCGTTTCAAAAAACTGACTGATATAAATAAAAACTCTTC 5000

G C G T K N V Y K P Q C P R C L A R K I S V S K N -

**ORF 2** - K N I R F K K L T D I N K N S S

GCAAATTTCCTCCAATTCACAGACTATATCACATTCCAACCTGAACGTTCAAATTTTAAATCAATCTAAAAACGAAGATTTATCCATTAATCGAATAATC 5100

Q I S S N S Q T I S H S N L N V Q I L N Q S K N E D L S I N R I I

TATCCTTATAAGCCGTACCATGAAAGATTAAGAAATTATATTGCTGTTCGTAACAGAATTTTTAACACTGATTCGTTGTCCGTTTGTCCGCTGAAAAAAC 5200

Y P Y K P Y H E R L R N Y I A V R N R I F N T D S L S V C P L K K P

CTAAACGATCCACAATACGTCTTAGACGTTATTTTAAATCTCGCAAATTAATATCTAAATTTGTAATATCTGCCATTATTAATAATGAAAAAGATAAGCG 5300

K R S T I R L R R Y F K S R K L I S K F V I S A I I N N E K D K R

ATATTATGCCAAAATCAAATTTTTAGAATTCGACGAGTATGGTCTCCTTGACACGGGTGCTAACGTATCGTGTATTGGCTCAGATTTGGCCACTTATAAT 5400

Y Y A K I K F L E F D E Y G L L D T G A N V S C I G S D L A T Y N

TTTGCTAATTGTCCTAATTTTCATCCCTTAAAAACCTTCGTGAAAACTGCTGATGGCACAATTCAAAAAACCATTGGTATGCTAGAAGTAAAAGTTTCAT 5500

F A N C P N F H P L K T F V K T A D G T I Q K T I G M L E V K V S F

TTAGAGATCAAGTTGAAAAAGTTAAATTTTTAGTAGTCCCATCGATTTCCCAACGAGTCATTTTAGGATTAGACTTTTGGAAGATATTTAAACTTGCGAC 5600

R D Q V E K V K F L V V P S I S Q R V I L G L D F W K I F K L A T

AGAGATTTTCGATTCTGCTATTGTGTCAGATCCGTCCAAGTTGTCAGATTCACAAAAACCGTCCGATATGTTGTTCGAGCCTTTAGAGGAAACTAATCTG 5700

E I F D S A I V S D P S K L S D S Q K P S D M L F E P L E E T N L

ACCAAGGAACATAAATACCCCCTTACCACCACACAGCTTCAGCATTTAAACATTATAATAAACCTTTTTCCAAACTTTGAGAAGCAAGGTTTAGGCAAAA 5800

T K E H K Y P L T T T Q L Q H L N I I I N L F P N F E K Q G L G K T

CCACCCTAATACAACATGAAATTGATGTTGGTGATGCAAAACCCATCAAGCAAAGGTTTTACCCTGTTTCCCCTGCGGTGGAAAAATTAATATACAATGA 5900

T L I Q H E I D V G D A K P I K Q R F Y P V S P A V E K L I Y N E

AATAGACCGAATGCTCGAATTAGGGGTAATTGAAGAATCCACTTCTGCTTGGAGTTCTCCTATGCGATTAGTGCTAAAACCCAACAAAACTAGACTCTGT 6000

I D R M L E L G V I E E S T S A W S S P **/**M R L V L K P N **/**K T R L C

**Retrotranscriptase / I /**

TTAGACGCCCGGAAATTAAATCAAGTAACCAAGAAAGACGCGTACCCATTACCGAACATAGAAGGCATATTTTCAAGACTACCGAAAGCTAATATAATCT 6100

L D A R K L N Q V T K K D A Y P L P N I E G I F S R **/**L P K A N I I S

**II /**

CTAAACTTGATCTGAAAGACGCATATTGGCAAATCGGCCTCGCCGACCAGTCTAAGGCGCTTACAGCATTTACGGTACCAGGTAGACCATTGTACCACTT 6200

K L D L K D A Y W Q I G L A D Q S K A L T A F T V P **/**G R P L Y H F

**III /**

TGTTGTTATGCCTTTTGGTCTCTGTACAGCGCCTCAGACAATGTGCCGTCTTATGGATCAACTGATACCGCCAGATCTTCGTCATTGTGTTTTCGGCTAT 6300

V V M P F G L C T A P Q T M C R L M D Q L I P P D L R H C **/**V F G Y

**IV /**

CTCGATGACTTAATAATTGTGTCCGAAGATTTCCAGTCCCATTTAGCTACCTTAGTCAGAATCGCAGAACAATTTCGCAGGGCCAATCTCACATTAAATA 6400

L D D L I I V S E D F Q S **/**H L A T L V R I A E Q F R R A N L T L N I

**V / VI**

TTTCCAAGAGTGCATTTTGCGTAACTGAAGTAAAGTATTTAGGTTTTGTCATAGGGCAGGGAGGAATCAAAACAGACCCACAAAAAGTGGAGGCAATCCT 6500

S K S A F **/**C V T E V K Y L G F V I G **/**Q G G I K T D P Q K V E A I L

**/ VII /**

TAAATGGCCGACCCCGAAAAACCTTAAACAAGTTCGCGGATTCCTAGGCATCGCCGGTTGGTACCGCCGATTTATCGACAATTTTTCTACCGAAGTATAC 6600

K W P T P K N L K Q V R G F L G I A G W Y R R F I D N F S T E V Y

CCTATAACGGAGGTCCTGTCGACAAAACGAAAATTTAACTGGACACCAGAAGCACAACAAGCTTTTGAGAAAATCAAGTCGTTGCTCACAACAACTCCAG 6700

P I T E V L S T K R K F N W T P E A Q Q A F E K I K S L L T T T P V

TTCTGTCAAATCCAGATTTCACGAAAAAGTTCTATTTGCACTGCGATGCAAGTGACTTTGGAATAGGTGCGGTTTTGGTTCAATTGGACGAAAATGGATC 6800

L S N P D F T K K F Y L H C D A S D F G I G A V L V Q L D E N G S

GGAAAAACCTATCGCCTATATGTCCAAAAAACTTACCACAGCTCAGCGCAATTATAGCGTAACTGAGAGGGAGTGTCTAGCTGCTATTGAAGCCATCAAA 6900

E K P I A Y M S K K L T T A Q R N Y S V T E R E C L A A I E A I K

AGGTTTCGGTGTTATCTAGAACTCCAAGAGTTTGAGGTCATTACCGACCACTCCTCTCTAGTATGGCTAATGAAGCAGCCAGATCTGTCGGGAAGATTAG 7000

R F R C Y L E L Q E F E V I T D H S S L V W L M K Q P D L S G R L A

CCAGGTGGGTCTTTAAACTTCAACCATACAATTTCACAGTAAGCCATCGTAAGGGAAAATACCATATAGTTCCGGACGCCCTAAGTAGAATCCAGTATGC 7100

R W V F K L Q P Y N F T V S H R K G K Y H I V P D A L S R I Q Y A

AGAAATCTCTGATATACATCTGTTGGAACCAGAAATAGATTTGCACTCACCACATTTTCAGGATCCAGATTACCAAGAACTTAAGAAGAAAATCTCTGAA 7200

E I S D I H L L E P E I D L H S P H F Q D P D Y Q E L K K K I S E

AATGCTGTAAAATATCCTGATGTTAAAATCGTTGAAAATTTTGTATATATTCGAACTTGTCATTCTACTGGAGATTTAGATGGAGACCAAAGATCATGGA 7300

N A V K Y P D V K I V E N F V Y I R T C H S T G D L D G D Q R S W K

AACTATGGGTACCATTGCAGCTAAGACAAGAGGTTATTAAAAAGACGCATGAGTCCATAACAATCCATGGCGGTATGTCAAAAACACTGGATTTAATAAG 7400

L W V P L Q L R Q E **/**V I K K T **H** E S I T I **H** G G M S K T L D L I R

**Integrase / Zinc finger domain**

ACGAAATTTTTATTGGCCAGGCCTGGTAACTAATGTTCGTGATTTTGTTCGTGATTGTGAAATCTGCAAACAAACAAAAGCCCCAAACACGATTTTAAAA 7500

R N F Y W P G L V T N V R D F V R D **C** E I **C** K Q T K A P N T I L K

CCTAAAATGGGCCAGCAATCTGTTTCATTAAGACCCTTCCAGAGATTATATTTAGACATATTAGGACCCTATCCCAGAAGCAAAAATGGTCATATAGGGC 7600

P K M G Q Q S V S L R P F Q R L Y L D I L G P Y P R S K N G H I G L

TATTTATAGTATTAGACCATTTTTCTAAATTCCATTGGCTTTGTCCTCTTAAAAAATTTACATCTAAGTCTATACAAAGTTTTCTTCTAAGTAACATAAT 7700

F I V L **D** H F S K F H W L C P L K K F T S K S I Q S F L L S N I I

**DDE domain**

TCACGTCTATGGTGTACCTGAATTTGTTGTAACTGATAACGGATCGCAGTTTAGGGCAAATGACTTTGAAGTGTTTTTGACCAAATTAGGAATTAAGCAT 7800

H V Y G V P E F V V T **D**  N G S Q F R A N D F E V F L T K L G I K H

ACCCTAACCGCTTTGTATTCCCCACAGTCAAACGCGTCAGAACGCGTAAATCGTTCCATAATATCAGGAATTCGTTCATTTTTGAAACAGGATCATAGAG 7900

T L T A L Y S P Q S N A S **E** R V N R S I **/**I S G I R S F L K Q D H R D

**/**

ATTGGGACGAAAATATTAGCTCTATAAGTTGTTCGCTTAGAAATACTTATCACCAAACTATAAAATGCTCTCCATACCATACCTTGTTTGGATTGGAAAT 8000

W D E N I S S I S C S L R N T Y H Q T I K C S P Y H T L F G L E M

GATAACTCATGGCTCATCCTATGAATTGTTGAGAAAATTAAAACTTTTAGATGAATCAGTGAATCCCCTTCCCAGAGAAGATAGTTTGGCATTGCTCAGA 8100

I T H G S S Y E L L R K L K L L D E S V N P L P R E D S L A L L R

AAAAATATTAGAAAAAACATACAAGATTCATATGAAATAAATGAACATAATTATAATTTAAGAGCTAGGCCAATACAATACAAGGTTGGACAGGAAGTTT 8200

K N I R K N I Q D S Y E I N E H N Y N L R A R P I Q Y K V G Q E V F

TCCGCAGAAACTTTTCTCAAAGTTGTTTTGCTAAAAATTACAATTCCAAACTAGCACCAATGTTTCTGAAGGCAAAAATTCGCGAAAGAACCGGAAATAA 8300

R R N F S Q S C F A K N Y N S K L A P M F L K A K I R E R T G N N

**PPT**

CTGCTACATATTGGAGAACAC**AGAGGGCAAGGTTAT**CGGAACTTATCACGCAAAAGACATTCGACCTTAATAATCGTTTTTCCGTCTAATTTTGTCGTAT 8400

C Y I L E N T E G K V I G T Y H A K D I R P -

ATATACCAAAATTATCTGGTTTGTAATGGACGATTATTGTTAAAAAGTTTAATTCTTGAAAGTACATATTTTATATTGTGATACATATTTATATATATAA 8500

**LTR**

ATATGTATATAAACAAATATGTATATGTTTGGTTTTTATTCCACATTCAAATTAAAATTTTGCCTCAAATTCGGTTTTTTCGAAAATTTCGTTTTGAATT 8600

TTAACAGTAATGCTTTGTGTGTTGTATTTCACCTCACACTGTTGCATTTACTTTAAAAAATCAAGTTAGCCACATTGAAAATAGCCACTTTGTTTTGACA 8700

CATTGCAACGCATGAGAACGCATAGCAACGCATATTAACGCAACTAAAAAAAATAGAAAAATCCCGAACACTAACGAGAATTGAAGCATGGTTTTTCGTA 8800

ACCAAATGTGTTAACCTTCTTTTGATTTTATACCTCAAAGTGAACACATCGTTCAGTTAAAATATATAATATCCTTTGTACAGGACACACTTTATTTTTT 8900

CTACCAATTTAATTTTAAAGTTTCATAAAGGTAAATCTCGCAGACTTAAAGATTTCGATGTAGTTAATTAACATACAATATTTTTTTAATAGACAACTAG 9000

AGTGGAAATTACCATATTGCTAAATCCCAAGATTCAACATAACCTGAATCCTTCTTAATCCTTGAGGTTATTGGAAGACTAATAAATTGTGGAAAAACAC 9100

CATTAAAAGCCTCGAATATACAAGCTAATTTCCATCTAAAAAGGCACGGTGTGTTTATTTAGCCACCACTGTATCCACTTTTAAGTCACATAAAAAAAAC 9200

CTAAAAACCACGCTCACAAGAATTGTGCTTACCGCTGATCATCATTATTTTAAATCTACCGATAAGACAACACCTAAAGTCAAGTGCTTCATTATTGTGT 9300

ATCAGCCAAAAAGAAATTCGAAAAAAAAACTACAAATTTTATAAGCAAATATTAAGAGTCTCAGACGAGCTTCAAAACACCTACCAGCTTCCAAAATAGG 9400

ACCGGATAAGTCGTCTAGGAAAACCTCCAAAAATCATCTAAGTTCTTCATAAGACAATTACCCAAATTTGAGTCTCAAAATAATTAAAATTTTACGTAAG 9500

ATTGTGCATGTTTAAGTTTTTTTTTTGTGACTTTATATTCTTTTTTTCTTCTTAAAATCAAAAGGTGCAAACAATTTAAATTACCTTTAATAAATCAAAC 9600

TACTTGAATTTCTTAACAAAATTTATAAGTGTGATTAAAAACTTACAACTAAACCAATAAGAAAAATCACCTAACTTAAATCTAATTTAAATATATATAA 9700

CAAATTAAAAACCAAAAGTTTAAAATGTATAAATGTAAAATATAAAGATTAAAAGCAAAAAAAAAGAAACTTAACCTATATTGCACATAAATTATCACAG 9800

TTGAATTATTTATTAATTAAGTTCTTTTTATATATAATATAATTAATGACATGCAAAACAAATAAGATCTATGTAACAACAAAGTAAACATCCAACAAAA 9900

GCAGTCTGACGAGGCCTCATCATTCAGCGTTTCCAGAATTAAGTTGGTGAGTTTTTTTTCTCTGTTTTAAGTAATATTATTAATTAAAAAAATTTAGTTT 10000

TGCATTCATTATTATTAAATTTAATAACAAAATCAGAATATCTGTAATTTAAAAGAAACAATAATCAAATTTTTGAGGTTTGCTTTTACATATTCCTTGA 10100

TTTGCTAGGGAATACTGTGTCAGTAACAAAGTCAAAAAAGAACAAGAAATAAAACACATTTCTTTGTTTTCTCACGCTTTTTTTTTTTTGTTTATGCACA 10200

AGTAATTCATAAATTGATATGTAAAAGCAAAACACAAAAAATCGAAAACCATAAAACCATAAAACAAAAACAGTTTAAGCAATGATCATTTAACATTGTA 10300

AATGATCATTACAAGATACCCATTGTATTCTCTAATTTGATTATTCTTTTTTTTTACTACAGTATTGTCCCAAAAATATAGAAATATAGTTTACTAGATT 10400

ATTTCTTTAATATTATTATAATTATCTATATTTAATTACTAGGCATTTCTTTTCTTAACAATTTACTTTTACTATTGTTGGTTAGAAAAAAAAAAATGTT 10500

ATAATAATGTTTTTTCTAAATTTTGATCTTGTAAACCAAATTTATATTTAAAAGTCATTGAATTTTTTTTATTACCTACCTAATGGTTTTCCTCAATTTT 10600

GTTA**AATAAA**TTGTTTACTTGAATTATGAAATTCTAAGTTATTTTTAATGTTTTCGCGATGAGATGAAAAAGTAAGTGTGTATTTAATTAGGGATCTTAG 10700

PolyA

TGTAATATTCGCCAATAAGATCATTGGATGGGAGGGTTTAGAGGCGATTATCCCAAACAACATCATGGGATAATAATAAAAGAGTTTTTCAATTAGTAAG 10800

TTCAGTCAGGGAGTCTATAATAAATTATTTATGTATAATGATTCCTATATGAGAGAAAATTTAACATGAACAAGAATCCACCCTCCGACTGACCGCTAGT 10900

GGCAATTAATGGCTCCCGTCGTTCATTTAAATTTTAATCCAAAAGCTGTAACCTCATATAGCGTTGAATATGTATTTATTCAATTGTACATTACA 10995
