# Supplementary material for: Diversity of transposable elements and repeats in a 600 kb region of the fly Calliphora vicina
Source: Mob DNA. 2013 Apr 3;4:13. doi: 10.1186/1759-8753-4-13 (PMC3630058; doi:10.1186/1759-8753-4-13)
Supplement: Additional file 4: Figure S2 — CsRn1_Cv1. Full nucleotide sequence of the CsRn1_Cv1 element of C. vicina and protein translation of the two ORFs. Nucleotides in red are LTRs, in bold and underlined PBS and PPT sequences. Amino acids: Nucleocapside CCHC domain in red, protease in pink, retrotranscriptase in blue, RNase domain in green, and Integrase in pink. [file 1759-8753-4-13-S4.doc]

TGGGAACACATATTTAATATCTAAAATCTACTTGTTAAAACTTTTAATTTCAAATTTTGTATAATAATATTTTAATCATTTTTAGCTTTGAATTTTGAGT 100

**LTR** **PBS**

TTTTAAGTTTTTATAATTTTCAATAAATAATTTAGTTTTCTTTTAACTTTTCTAAATAAATGTTGTTTTATTAACTACATTTAATTGG**TGACCTCGCGTT** 200

**TTTTG**AACATTTTCAAACTATATTTATTGAAATGTCTTCTCCTGAGAGAACAGCTTCAACAAGCAATATTGGAGATGCTCCAAGTGCATATATTGCTGCT 300

**ORF1** M S S P E R T A S T S N I G D A P S A Y I A A

TCAAGTATTCAAAAACTGCCACCTTTTTGGAAGGAGAATCCCAAGCTATGGTTTATGCAAATTGAGTCTATATTTGGAATTTCTGGTATTACACGAGATG 400

S S I Q K L P P F W K E N P K L W F M Q I E S I F G I S G I T R D E

AAACCAAGTTTCAGTATGTTATTGGGAATATTGATTCAAATTTTTTACCACATATTTCGGACATTTTGGAATCACCTCCATCTGATGGCACCACTAAGTA 500

T K F Q Y V I G N I D S N F L P H I S D I L E S P P S D G T T K Y

CCAAGCTGTTAAGGATCGCTTAATTGGAACCTTTTCCGAATCTCAAGAGTCTAAACTTCGCCGACTTTTGAAAAGTCATCAACTAGGCGATCAGAAACCC 600

Q A V K D R L I G T F S E S Q E S K L R R L L K S H Q L G D Q K P

TCGCATTTCCTTCAAATTATGAGAAATATTGCTTCTGGTCAACTTAATGATTCATTACTCAAAACTCTTTTTTTGGAACAATTACCTGAAGATGTGAGAT 700

S H F L Q I M R N I A S G Q L N D S L L K T L F L E Q L P E D V R S

CAATTCTCGTTATTAGTCAAGTAAGTGATGTTAGTGCATTGGCACATCAAGCCGATAAAATTATGAGTCTACGTTCACCACAACTTTTTGAGATGAAATC 800

I L V I S Q V S D V S A L A H Q A D K I M S L R S P Q L F E M K S

AACTCCTGAAAATGCTGATATATCAATGTTATGTCAAAAGATCGATGCATTAGAGAAGAAAGTTAACAGCTTTACTCGTAGTCGCAGTACTGAACGACAA 900

T P E N A D I S M L C Q K I D A L E K K V N S F T R S R S T E R Q

AATCGAAACTCTTCAATATCTGGTAAGTCAAGAAATCGAAGCAAATCTCGAAATAAAGATTGGTGCTGGTACCACAACAGATTTGCTGAGAAGGCAACCA 1000

N R N S S I S G K S R N R S K S R N K D W **C** W Y **H** N R F A E K A T K

**Nucleocapside: CHCC domain**

AATGCATCAAACCCTGTTCTTTTTCAAAAAATTAAAATCTCATCCGGATATTGCGACATTTTCGGATGATCAAGTTAACTCATGTCGCTTATTTGTAAAA 1100

**C**  I K P **C** S F S K N -

M H Q T L F F F K K L K S H P D I A T F S D D Q V N S C R L F V K

**ORF 2**

GACAACGCATCGAAAAAATGTTTCCTTATTGATACTGGTGCAGATGTATCAGTCATCCCTCCGAGTTTTCACATTCCTATCAACTCGTCAAATCTAGGCA 1200

D N A S K **K C F L I D T G A D V S V I** P P S F H I P I N S S N L G I

**Protease**

TAAAAATTTTTGCTGCTAATGGTTCACAAATTCGTACTTTTGGTACTAAAATTCTACACCTGGACTTAGGACTTCGTAGAAATTTTTCGTGGAATTTTAT 1300

K I F A A N G S Q I R T F G T K I L H L D L G L R R N F S W N F I

AATTGCAGACGTGTCTAAACCCATAATAGGATCTGATTTCCTAAAATATTACCATCTTCTTCCAGATCTTAAGAGAAAAGCTTTGATAGATGGTACAACA 1400

I A D V S K P I I G S D F L K Y Y H L L P D L K R K A L I D G T T

TTATTACATGTCTCCGGAAAGCTTTTAGAATCTTCGTCTCTGGGAATCAAAGTATTAGTTCATGATGACAGTGTGTACAACAAACTTTTATTGAACTATC 1500

L L H V S G K L L E S S S L G I K V L V H D D S V Y N K L L L N Y P

CAGATGTCTATCAAGCTACAGCAATTCCCGGAATAAAAAGCAAACACAATATTTATCACCATATAGAAACAACTGGACCACCCGTTTTTGCTAAGGCTCG 1600

D V Y Q A T A I P G I K S K H N I Y H H I E T T G P P V F A K A R

TCGTTTAGATCCTAATCGTCTTCAAATTGCCAAGAAAGATTTTGAATTTTTACTCCAGTTTGGTATTATTAGACCGTCAAAAAGCAATTGGTCGAACCCG 1700

R L D P N R L Q I A K K D F E F L L Q **/**F G I I R P S K S N W S N P

**Retrotranscriptase / I**

CTTCATATGGTCCCTAAGAAAAATGGTGAGTGGAGATGTGTAGGTGATTATCGATTTTTAAATAAAATAACAGTGCCGGATCGGTATCCAATACCATTTA 1800

L H M V P K K N G E **/**W R C V G D Y R F L N K I T V P D R Y P I P F I

**/ II**

TCACCGATGCCAATGTTAATATTGCTGGTTGCAGTGTATTTTCAAAATTGGATCTTGTTCGTACATTTTACCAGATTCCTGTTTATCCTGATGATATATC 1900

T D A N V **/**N I A G C S V F S K L D L V R T F Y Q I P V Y P D D I S

**/ III**

TAAAACCGCTGTTATAACACCTTTTGGATTATACGAATTTCTTAAAATGCCATTCGGACTAAGGAATGCTGCTCAAAGCTTTCAAAGGTTAATGGACGAA 2000

K T A V I T P **/**F G L Y E F L K M P F G L R N A A Q S F Q R L M D E

**/ IV**

GTTCTTCGTGGATTACCATTTGTCTTTGTTTACATTGATGATGTCCTTCTTTTTTCTAAATCAACAACTGAACATCTAGAGCACCTAAAAATAATTTTCG 2100

V L R G L P F **/**V F V Y I D D V L L F S K S T T E **/**H L E H L K I I F D

**/ V /**

ATCGATTTCAAGAATATGGAATTGTTGTAAATCAAGGTAAATGTAGTTTTGGTAAATACGAAATAGATTTTCTTGGATTCCACATATCGTCCGATGGTAT 2200

R F Q E Y G I V V N Q G K C S **/**F G K Y E I D F L G F H I **/**S S D G I

**VI / VII /**

TCTTCCCACTCAAAGCAAAATTGAAGCCATTCAAAACTTTCCTAAGCCAGAGTCAATCAAAGATTTAAGACGTTTTCTTGCTATGATCAACTTTTACAGA 2300

L P T Q S K I E A I Q N F P K P E S I K D L R R F L A M I N F Y R

CGCTTTCTTCCTCATGCTGCCATAGAGCAAGCACCACTAAATAATCTTTTGAAAAACTCTGTTAAAAATGATAAACGTCCTGTTCCATGGTCTTCCCAAA 2400

R F L P H A A I E Q A P L N N L L K N S V K N D K R P V P W S S Q T

CTGAAGAAGCCTTTGAGAAATGTAAGTCAAGCCTTTCTAATTCTACATTATTATTTCATCCTATAAGTGATGCCGAATTAGTTGTAAAAGTTGATGCATC 2500

E E A F E K C K S S L S N S T L L F H P I S D A E L V **V K V D A S**

**RNase H**

TGATTTTGCTATTGGCGCTGTGTTGGAACAAAAAGTTGGTAACGATTGGCAACCATTATCATTTTTTACAAAGAAATTAACGGATACTGAGAAACGGTAT 2600

**D F A I G A V L E Q K V G N D W Q P L S F F T K K L T D T E K R Y**

AGTACCTATGACCGCGAATTGTTGGCAATTTATGCTGCCATTAAATATTTCCGTGATTATGTTGAAGGTCGTGAATTTTCAGTACATACTGACCACAAAC 2700

**S T Y D R E L L A I Y** A A I K Y F R D Y V E G R E F S V H T D H K P

CTCTCATTTTTGCCTTTTCTCAAAAGCCTGAGAAAGCAAGTCCCCGCCAACTTCGACAATTAAACTTCATTGGTCAATTTACTACAAATTTTAAACATGT 2800

L I F A F S Q K P E K A S P R Q L R Q L N F I G Q F T T N F K H V

CAAGGGCACCGAGAACGTTGTAGCTGATGCTTTTTCGAGAATCGAATCAATATCTCTTCTTGATTATGATGAACTTTCAAAATTACAGAATAAAGATACG 2900

K G T E N V V A D A F S R I E S I S L L D Y D E L S K L Q N K D T

GAGCTTCAAAATCTTTTGAATTCCAACACGTCTCTCAACTTGCAATTAGTTTCCATTCCTGGTACTAAAACAAAAATATTTTGTGATGTATCCACCGATA 3000

E L Q N L L N S N T S L N L Q L V S I P G T K T K I F C D V S T D N

ACTTTCCTAGACCGTACATTCCAAAGGACTTAAGAAAATCAATATTTAATGGATTACATAATTTGTCTCATCCTGGCGCAAAAACATCAACCAAGCTCGT 3100

F P R P Y I P K D L R K S I F N G L **H** N L S **H**  P G A K T S T K L V

**Integrase: Zinc finger domain**

TACTGAACGATATATTTGGCCATTTATGAAACGCGATTGCAGATATTGGACGAAAATTTGTCAAGCTTGTCAAAAAGCAAAAATTTCTCGTCATACTAAA 3200

T E R Y I W P F M K R D C R Y W T K I **C** Q A **C** Q K A K I S R H T K

AGCCCTATAGGACATTTTCCTCTAGCTTCTCGCCGTTTCGCAGAAATTCATATTGATTTAGTTGGTCCCCTACTTCCATCAAATGGTCAACGATATTTAC 3300

S P I G H F P L A S R R F A E I H I D L V G P L L P S N G Q R Y L L

TAACTTGTGTCGATCGATTTTCACGTTGGATGGAAGCTTTTCCAATGCCAAACTGCTCATCAGACACAATATGTGAAACTTTTATGAATGGATGGATATC 3400

T C V **D**  R F S R W M E A F P M P N C S S D T I C E T F M N G W I S

**DDE domain**

AAGATTTGGTTCACCTGATGTAATTCACACTGATAGAGGACGACAATTTACATCAAATAATTTCCGAAGTTTAACACAATTTTTGGGAACAAAAATCAAA 3500

R F G S P D V I H T **D** R G R Q F T S N N F R S L T Q F L G T K I K

TTTTCAACATCATATCATCCCCAATCGAATGGTTTAGTTGAGCGGTTCCACAGAACATTAAAAGCTTCAATTTCCTGCCATGAGGAATCACAATGGACAA 3600

F S T S Y H P Q S N G L V **E**  R F H R T L K A S I S C H E E S Q W T K

AAGTTTTACCATTGGTTTTGTTGGGACTACGTGCATCAGTAAAGGATTCAATACATTGCTCTCCAGCAGAAATAGTTTATGGTGAAATTCTTCAACTTCC 3700

V L P L V L L G L R A S V K D S I H C S P A E I V Y G E I L Q L P

TGGACAATTCTTTGGCAAGACTACATCCTTAATCAACAGTACTGATTTTGTAGCACAACTGCAAACAATTATCTCAAGAATCAAACCAACATCCAGTTCT 3800

G Q F F G K T T S L I N S T D F V A Q L Q T I I S R I K P T S S S

AACAACACCAAACCATCTGTTTTTGTCAACAAGGATTTACAAGAATGTACTCATGTCTGGATAAGGAATGATCTCATTTTAAAACCACTTCAACAAATGT 3900

N N T K P S V F V N K D L Q E C T H V W I R N D L I L K P L Q Q M F

TTCATGGTCCTTATAAAGTTATTGCAAAGTTTGACAAATATTTTACCCTTCAAATAGGAAACAAGATCGATAACGTATCAATTGATCGTTTAAAACCTGT 4000

H **G P Y** K V I A K F D K Y F T L Q I G N K I D N V S I D R L K P V

**GPY domain**

TTTTTTAGAAATTGAATGTAATGATGTTCAGGGTAACAAGAATGATAATGTTGCAGATATTACTTATAAACGAGAATTAAAAACAGTATCGTTTTCACT**A** 4100

F L E I E C N D V Q G N K N D N V A D I T Y K R E L K T V S F S L

**PPT**

**GGAGGGGAATAATG**TGGGAACACATATTTAATATCTAAAATCTACTTGTTAAAACTTTTAATTTCAAATTTTGTATAATAATATTTTAATCATTTTTAGC 4200

G G E - **LTR**

TTTGAATTTTGAGTTTTTAAGTTTTTATAATTTTC**AATAAA**TAATTTAGTTTTTTTTTTAACTTTTCTAAATAAATGTTGTTTTATTAACTACA 4294

PolyA
