# Supplementary material for: Diversity of transposable elements and repeats in a 600 kb region of the fly Calliphora vicina
Source: Mob DNA. 2013 Apr 3;4:13. doi: 10.1186/1759-8753-4-13 (PMC3630058; doi:10.1186/1759-8753-4-13)
Supplement: Additional file 5: Figure S3 — Pao_Cv1. Full nucleotide sequence of the Pao_Cv1 element of C. vicina and protein translation of its ORF. Nucleotides in red are LTRs, in bold and underlined PBS and PPT sequences. Amino acids: RING finger domain in light green, retrotranscriptase in blue, Pao peptidase in dark green and Integrase in pink. [file 1759-8753-4-13-S5.doc]

TGTTGCCGACAGAATTTAAAATGTTAGGTCAGTTAAAAATTATACACTAGAAAAACAAATGTTAATTTAATAATAACTTTTGTTGCAACTATGAATATAA 100

**LTR**

AATTCCATTTTCTAACACTGTAAAATTGTATGTATGTATATATAAATGATTTATAGGATACATAATCAAAAATCACGTTTTGTAGCCCATATTACAGCAT 200

AATAAAACATTAAAATTTTGAAACTAATTATTGAAAACTAAGGTAATATAAAACTGAAACTGAATTAAAAATAACTGAATTAATAAAGAGTTCGCTTTCA 300

GAATTTTAAAGCGACACATTAGTTTGCTGTTTAAAACTTGGTTTTTTATTTTACAAATCTTTAAAATTCTTATTAAAACAAGATTATGATGCAAGGTGGA 400

M M Q G G

AATTTGAATTCAAGCTGCATATTATGCACGGATCCCGATGACGGAAATATGGTTACGTGCTCCAAATGCCTTGGTAGTTATCATTATACTTGTGGGAACA 500

N L N S S **C I L C T D P D D G N M V T C S K C L G S Y H Y T C G N I**

**PHD zing finger domain (RING finger)**

TACATGACAGCGTAGAGTTTTTTACTTGGATCTGTCAAAACTGTTCTGTACAACAACAATATATTTCTGAACCTAACCTCATTCAGCTGGGCTCACGACA 600

**H D S V E F F T W I C Q N C** S V Q Q Q Y I S E P N L I Q L G S R Q

AGAGATGAATGCTACTGCAACACAGCCAGCCGATCCATTAACAAGACGTCGAGATACCGCCAATTCCAATGCTGCACAATCTTCTTCCTTGCATTTGAAT 700

E M N A T A T Q P A D P L T R R R D T A N S N A A Q S S S L H L N

ATAAGCGCTGAAAAACAACTCCAGTTGCAGCAGCTTGAAGAGGAGATGCAATTGAGAAAAGAATATCTGCAACGAAAATACGATTTGCTTCGTTCTAATA 800

I S A E K Q L Q L Q Q L E E E M Q L R K E Y L Q R K Y D L L R S N N

ATGAGAGTCAATCGATTCTTCACTGCGGCCAGATAACAAATGCATGGCCAGTGGCAGGCAATAATAATAATTGTAGTAGTTCACAGAGTATGCAAAATTG 900

E S Q S I L H C G Q I T N A W P V A G N N N N C S S S Q S M Q N C

TTATCAAACAGTGAGTATAAATAGAATGCAAAATAACTCTCAAAATATAAATCCACAATCACGCGGCAATTGTCAACAAACAAACATGTCTCAATCTCCT 1000

Y Q T V S I N R M Q N N S Q N I N P Q S R G N C Q Q T N M S Q S P

AATATAATTAACAATGACTCACATCGTCATTGTGAATATTCAACACAAACAATTCCCAATATTCCTACACCTCAATGCCACAGTACAAATATTAACCGCG 1100

N I I N N D S H R H C E Y S T Q T I P N I P T P Q C H S T N I N R A

CTAATATGACATTTGTAAATGATCGCCCACTGAACATACCTCACACCAACAATCAAAACAACTTTGCAGCTGCTTATCAAAATACAAATTTGTTTACAAA 1200

N M T F V N D R P L N I P H T N N Q N N F A A A Y Q N T N L F T N

TGAAAATATTACCAGCAATAATTTTAATAATATTACAACAACACAACTGGTTGCTCGACAGTCTATTTCTAGAGACTTACCAGCCTTTAATGGTGACCCC 1300

E N I T S N N F N N I T T T Q L V A R Q S I S R D L P A F N G D P

AAAGAGTGGGCAATATTTATCAGTGCATTTGAGCAGAGTACCCGGGTTGCTGGTTATAGTAATGAAGAAAATCTTATTAGATTACAGAAATGTTTGCATG 1400

K E W A I F I S A F E Q S T R V A G Y S N E E N L I R L Q K C L H G

GCAAGGCACGGGAAGCTGTTCGAAATTGTTTGATTTTACCCGATATGGTTCCGGACATCATCAGGACATTGAAAATGTATTTTGGTAGACCAGAGTGTGT 1500

K A R E A V R N C L I L P D M V P D I I R T L K M Y F G R P E C V

GTTAACAAATTTAATTAATGAAGTTCGAAAACTTTCACTTCAAAGGGGTAAACTTGAATCTTTAGTAGAATTTGCTTTTGCTGTCAAAAATATATGCGCC 1600

L T N L I N E V R K L S L Q R G K L E S L V E F A F A V K N I C A

ACCATACGTGCTTCAAAATTAGAGGACTATTTAACTAATCCCACTCTATTGCAGGAACTGGTGGACAAATTACCAACCGATACTTTACTGCAGTGGGCTA 1700

T I R A S K L E D Y L T N P T L L Q E L V D K L P T D T L L Q W A M

TGTTTTCTAAAGATATTGCTCGCCCAAATTTGCTGGACCTTTCAGACTGGTTGTACGGCATAGCGGAGGCTACATGCCGTGTTACTATTCCAGTATTCGA 1800

F S K D I A R P N L L D L S D W L Y G I A E A T C R V T I P V F D

CAGCACCAATAAGTCATCTAAACAGGGACGTCTTAATACACACACAAACTCGGCACAACAAAAACCACCATATCAGTGCGTAATCTGCAACGAAGATCAC 1900

S T N K S S K Q G R L N T H T N S A Q Q K P P Y Q C V I C N E D H

AAAATTGCTCAGTGCTCTAAATTTTTAGACATGACAGTAACGGAGAGATGGGATATTGTTAAATCCAAAAATTTATGTCGTATGTGTCTGGGTAAGCACC 2000

K I A Q C S K F L D M T V T E R W D I V K S K N L C R M C L G K H R

GACGTAGGTGTTGGTTTCAAAAGCCTTGTGGAGAGAATGGGTGCATCGTAAATCACAATTCGCTACTACACAACAACGAGCAACCATCTGGTAGCACTGA 2100

R R C W F Q K P C G E N G C I V N H N S L L H N N E Q P S G S T E

AAGCATACTCAATAGTCACAAATCGATTGATGAATCATTTTTTCGCATTGTCCCAGTAACTTTGTATAATGACAAAAAATCTGTAAGCATATATGCTCTA 2200

S I L N S H K S I D E S F F R I V P V T L Y N D K K S V S I Y A L

ATGGATGACGGGTCATCTCTTACCCTTTTAGAAAAGGAAGTAGCGGACGTTTTACAGGTGTGCGGTATTAGAGATCCCCTTTGCATTCGGTGGACTGGTG 2300

M D D G S S L T L L E K E V A D V L Q V C G I R D P L C I R W T G D

ACATATCGCGATACGAAAACAATTCGCAACGACTTAATCTAGAGATTTCATCCACGAAACCAAATTCTAAAATATTTCCTATTAACAATGTCTATACTGT 2400

I S R Y E N N S Q R L N L E I S S T K P N S K I F P I N N V Y T V

GCAAAATTTAAATTTATCTGCAGAGCATATGATTGTAGAAAAAATAAAAGAAAAATATCCGTACCTTAAAAATATTCCACTTGAAGGATACCACAATGTT 2500

Q N L N L S A E H M I V E K I K E K Y P Y L K N I P L E G Y H N V

GTTCCATCAATGATTATTGGTGTAAATAATCCAAATTTAATAACGCCAACACAGATCTGTGAGGGTGGCTGGCAACAACCGGTAGCTTGTAAAACTAGAT 2600

V P S M I I G V N N P N L I T P T Q I C E G G W Q Q P V A C K T R L

TGGGTTGGACACTATTTGGTGGTGGCAATTCTAATATAGGCGGAAAACTGAATTATCATAAATGTGTTTGCAGAAATGATGATGAGATTCATAATATTAT 2700

G W T L F G G G N S N I G G K L N Y H K C V C R N D D E I H N I I

TAAACAATACTTTTCAGATGAAAATTTAGGTATTTTGCCACCTACAAAAGATATTTTCTCAAAAGATGATCAACAAGCTCTTGATATTTTAGAAAAAACT 2800

K Q Y F S D E N L G I L P P T K D I F S K D D Q Q A L D I L E K T

TGTAAATTCATAGACGGTCGATACGAAGTGGGCCTTTTGTGGAAGCATGACCAACCAGACTTACCAAATAGCTTTCAAACAGCTATAAATCGACTGCAAT 2900

C K F I D G R Y E V G L L W K H D Q P D L P N S F Q T A I N R L Q C

GCATTCAAAAAAAGGCTAAAAAAGATCCAGTGCTAGCTGAAAACCTCAAAAATCAAATTCTGAATCTGCAACAAAAAGAGTATGCTGTGAGATTACCAAA 3000

I Q K K A K K D P V L A E N L K N Q I L N L Q Q K E Y A V R L P N

TTGTGATTTAAAAGAAAGTGGAGGTAAAATTTGGTACTTGCCGACGTTTATTGTAAAAAACCCCCACAAACCCGACAAAATTCGTCTCGTATGGGATGCC 3100

C D L K E S G G K I W Y L P T F I V K N P /H K P D K I R L V W D A

**Retrotranscriptase I /**

GCAGCAAAATCTGGAAATTATGCGCTAAATGATTTTTTGACGAAAGGACCTGATTTACTTGTACCACTTATAAACATTTTATTTAAGTTTAGAATGGGCC 3200

A A K S G N Y A L N D F L T K G P D L L V P L I N I L F K F R M G P

????? /

CTATAGCAATATGCGGAGACATTGCTGAAATGTTTCATAGAATTAAAGTTCGAAAAGAGGATGCTTGTTCACAACGTTTTTTGTGGTGGGATGAAGATGG 3300

I A I C G D I A E M F H R I K V R K E D A C S Q R F L W W D E D G

/

TTCTCTACTTGTTTATCAATTAAATGTACTTACATTTGGGGCATCCTGCTCACCATGTATTTCGCATTATGTGCGGAATTTAAATGCGGAGAAATTTGCT 3400

S L L V Y Q L N V L T F G A S C S P C I S H Y V R N L N A E K F A

AGAAATCAAAAGGTTATTGATGCAATAACGAAACAACACTATGTTGATGATTTCATTGATTCAGCGAATACAGTTGAGGAAGCAATTGAATTGGCCTTAA 3500

R N Q K V I D A I T K Q H Y V D D F I D S A N T V E E A I E L A L N

/ /

ATGTTCGTGAGGTTCATGCTAAAGGTGGATTTTGTATGCGCAATTGGTCGTCGAATTCTGCCGAAGTATTAAGTGCATTGGGTGAAAGTAAGAATTTTGT 3600

V R E V H A K G G F C M R N W S S N S A E V L S A L G E S K N F V

AAACAGAACATTTGAATGCAACGAGTACTCATCTCAGTGGGAGAAGATTCTTGGCCTGTACTGGGACCCAAAAAGTGACGTATTTAAAATGAATTTAAAA 3700

N R T F E C N E Y S S Q W E K I L G L Y W D P K S D V F K M N L K

TTTGTCAGGCTCAAGCGTCCAATACTTAATTCAGAAATAATACCTACAAAAAGAGAAGTACTTCAAGTATTAATGTCTGTTTTTGATCCACTAGGTTTTG 3800

F V R L K R P I L N S E I I P T K R E V L Q V L M S V F D P L G F V

**Pao peptidase**

TGGCATGCTTTATGTCATATCTAAAAGTTATCCTCCAAGAGATATGGCGATCTGGAATTAACTGGGACCAACATCTTAACGAAGAGCTTTATAAGAAGTG 3900

A C F M S Y L K V I L Q E I W R S G I N W D Q H L N E E L Y K K W

GAAAAACTGGCTTAAATATTTACCAACAATTACTGCCGTTTCTATACCACGTTGTTACTCTCTAGCTTTACAACAAAACTTTATTGCGGAGTTGCACACA 4000

K N W L K Y L P T I T A V S I P R C Y S L A L Q Q N F I A E L H T

TTCGTCGATGCCAGTGAAGACGCTTATGCAGCAGTATCCTATTTTAGAATTGAGTATGCTGGTAAGGTAGAAATTAAATTTATAGCCGCAAAATCAAAGG 4100

F V D A S E D A Y A A V S Y F R I E Y A G K V E I K F I A A K S K V

TAGCTCCACTTCGACCAATCTCAGTACCAAGACTCGAATTACAAGCAGCGGTCATTGGTACAAGGCTGATGAGAACAGTCAGCTGCCACAATTTAAAATT 4200

A P L R P I S V P R L E L Q A A V I G T R L M R T V S C H N L K L

AAAAAGGAAACTCATGTGGACTGACTCAAAAACTGTTTTGAACTGGTTTAAGGGCGACCCACGAAAGTATAAGCAGTTCGTCATGTTCCGTGTTGCCGAA 4300

K R K L M W T D S K T V L N W F K G D P R K Y K Q F V M F R V A E

ATTTTAAAATACACAAATGCTTCAGATTGGAGGTGGGTTCCCAGTACAATGAACGTTGCAGACTATGCCACTAAATTTAGACCACCTAATGATAATTACA 4400

I L K Y T N A S D W R W V P S T M N V A D Y A T K F R P P N D N Y K

AGGATTGGTTTAATGGTCCCTCATGGCTAGAGAAATCTGAAAATGAGTGGCCAGGAAATTTAGATCCAGTTGATGAAGAAACTGTAGATGAATTAATTTC 4500

D W F N G P S W L E K S E N E W P G N L D P V D E E T V D E L I S

AGAAATTCGCCCACGTCATTTAAATATCCACACTCGTAAGTCAAATATAATCTCATTTAATTATCTATATTTTTCAAGTTGGTTTAGACTTTACCGAGCA 4600

E I R P R H L N I H T R K S N I I S F N Y L Y F S S W F R L Y R A

GTTGGTAATTGGTTGCTCTATATGGATCGCTGCTATCAGAAAGCTAAAAAACTGCAGATAGAAAATTCTTTGATTGTATCCCATCTTAATCGAGCAAAAA 4700

V G N W L L Y M D R C Y Q K A K K L Q I E N S L I V S H L N R A K N

ATATAATTTATAAGATAGTGCAGATGTCAGCCTTTCCAGACGAATATTTTGCTGTTAAATTTGGTAAAGAAATTCCAAAAGGCGGCGTTTGCTACAAACT 4800

I I Y K I V Q M S A F P D E Y F A V K F G K E I P K G G V C Y K L

TAATATCTACATTGACAACAATGGAATTATAAAAGTAAAAAATCGTGCTCAATATGCAACATCTGCTTGTGGTTTGCAACGAGATCTGATTATATTGCCA 4900

N I Y I D N N G I I K V K N R A Q Y A T S A C G L Q R D L I I L P

GGTAACCATCACATTACAAAATTAATAATTATAAATTATCATCTTGCGTTCCATCACTCAAATAACGAAACCGTTTTGAATGAAATTCGCCAAGTTTATT 5000

G N H H I T K L I I I N Y H L A F H H S N N E T V L N E I R Q V Y F

TTATCATAAAACTAAGAGTAGCTTACAAAGATGTACGAAAAAATTGTCAGTGGTGCAAAAATGAATTAGCGAAACCAATTCCACCTCAAATGGCACCAGT 5100

I I K L R V A Y K D V R K N C Q W C K N E L A K P I P P Q M A P V

ACCTCCAGCACGCCTTGGAGCCTTTCAGCGACCCTTTACATACGTGGGAACAGACTATTTTGGACCGATAGCAGTCACATTTGGAAGGAAGTCTCTAAAG 5200

P P A R L G A F Q R P F T Y V G T D Y F G P I A V T F G R K S L K

**RVE Integrase core domain**

CGTTGGGGTGTGATATTTACTTGCTTGACCATTAGAGCAATACACATTGAGATAGCTCATAGTCTTTCCACGGACTCTTTCCTTATGTGCTTAAGAAATT 5300

R W G V I F T C L T I R A I H I E I A H S L S T D S F L M C L R N F

TTATAGCTAGGCGTGGATCGCCAGCCGAAATATTCAGCGACAACGGAACAAATTTTCGAGGCGCTGATAGGTTTTTAAGGGATGAATTAAAAAACATCAA 5400

I A R R G S P A E I F S D N G T N F R G A D R F L R D E L K N I N

CTTTAATGAAGTACAAAGTAATTTGGCATATAAAGGAATATCTTGGAGGTTCAATCCTCCAGCAGCCCCTCACATGGGGGGAGCGTGGGAGAGACTAATA 5500

F N E V Q S N L A Y K G I S W R F N P P A A P H M G G A W E R L I

AGATCTATAAAAAATATTTTATATAAAATTTGCCCCACTCAAAAGTTTACTGATGAGAGTTTGAGAAGCTCACTAATGGAAGTTGAGATGATTGTCAATT 5600

R S I K N I L Y K I C P T Q K F T D E S L R S S L M E V E M I V N S

CAAGACCTCTTACTTATGTATCTATTGATTGTGCAGATCAAGAAGCAATTACTCCAAACCATTTTATTTTGGGAAGTTCAAATGGAACTAAACCGTTTTG 5700

R P L T Y V S I D C A D Q E A I T P N H F I L G S S N G T K P F C

TGAATTAGGCGACATAGATTACAGAATGTGCCTACGCCAAAGCGAGATATTTGCAAATGCCTTCTGGCGCCGTTGGGTTAAAGAAATGCTGCCAACCTTT 5800

E L G D I D Y R M C L R Q S E I F A N A F W R R W V K E M L P T F

ACCAGACGCAGTAAATGGTCACAACGTGTTAAACCAATTGAGGTAGATGATATCGTGCTGATTGTAGATGAAAATTCAAAAAGAAATACCTGGACTAAGG 5900

T R R S K W S Q R V K P I E V D D I V L I V D E N S K R N T W T K G

GGAAGATAGTTGAAGTGATAAAAGCAAGAGATGGTCAAGTGCGAAGAGCGAAGGTAAAAACTATCAATGGAGTTGTCGAGAGGCCTGCAGTGAAATTAGC 6000

K I V E V I K A R D G Q V R R A K V K T I N G V V E R P A V K L A

**PPT**

AGTACTGGACGTCGACAAT**AGAGTGGAGTAAAAC**CTCAAATAGAGCGCTTTACTGGGGGGAGAACTGTTGCCGACAGAATTTAAAATGTTAGGTCAGTTA 6100

V L D V D N R V E - **LTR**

AAAATTATACACTAGAAAAACAAATGTTAATTTAATAATAACTTTTGTTGCAACTATGAATATAAAATTCCATTTTCTAACACTGTAAAATTGTATGTAT 6200

GTATATATAAATGATTTATAGGATACATAATCAAAAATCACGTTTTGTAGCCCATATTACAGCAT**AATAAA**ACATTAAAATTTTGAAACTAATTATTGAA 6300

**polyA**

AACTAAGGTAATATAAAACTGAAACTGAATTAAAAATAACTGAATTAATAAAGAGTTCGCTTTCAGAATTTTAAAGCGACACATTAGTTTGCTGTTTAAA 6400

ACTTGGTTTTTTATTTTACA 6420
