# Supplementary material for: Diversity of transposable elements and repeats in a 600 kb region of the fly Calliphora vicina
Source: Mob DNA. 2013 Apr 3;4:13. doi: 10.1186/1759-8753-4-13 (PMC3630058; doi:10.1186/1759-8753-4-13)
Supplement: Additional file 6: Figure S4 — Cv-mar1 consensus sequence. Consensus sequence of the Cv-mar1 element and amino acid sequence of its transposase. At position 993 (shown in red) the consensus sequence has a T that gives a stop codon in the transposase; a third of the sequences have an A at this position, which would result in an arginine (R) residue. Underlined nucleotides correspond to the TIRs; the 5′TIR is incomplete. The blue dash close to the end of the sequence delimits a fragment found in one insertion only (see text for details). [file 1759-8753-4-13-S6.doc]

TAAAAATGCGGGATTTACAATAGATGGCGTATCTTTTGAACACGGTTTTTGTTTTTAATAACTTATATGTCATTTTGAAGGGTACATATCTGTCATTTAT 100

TIR

TCTCACTTAGTTATTGAACATTATAGTGTAAACAACAAAGTTTTTTTTTGCTTCGAAAATGTCGAATTTTGTGCCAACAAAGCGTCATATGCGGGAAGTT 200

M S N F V P T K R H M R E V

TTGCTTTACTTCTTTAATTTGAAAAAAAGTGCCGCTGAAGCACACCGATTGCTCACCAAAGCTTATGGTGAATGTGTTTCATCGGTTTCAACGTGCGAGA 300

L L Y F F N L K K S A A E A H R L L T K A Y G E C V S S V S T C E R

GATGGTTTGTGCGGTTCAGAAGTGGTGATTTTGACACGGAAGACAAAGATCGCCCAGGCCAGCCAAAAAAGTTTGAAGACCAAGAATTGGAGGCATTACT 400

W F V R F R S G D F D T E D K D R P G Q P K K F E D Q E L E A L L

CCATGAAGATTGTTGTAAAACTCAACAAGAGCTTGCAAAATCATTGGGAGCTACTCAAGCAGCAATTTCAAAACGTTTGCGAGCAGCAGGATTCATCCAA 500

H E D C C K T Q Q E L A K S L G A T Q A A I S K R L R A A G F I Q

AAGCAGGGAAATTGGGTACCATACGAATTGAAGCCGAGAGACCTTGAAAGACGATTTTGCATGTCCGAAATGATGCTTGAACGCTATAAAAGAAAATCAT 600

K Q G N W V P Y E L K P R D L E R R F C M S E M M L E R Y K R K S F

TTTTGCACCGAATCATTACTTGCGATGAAAAATGGATCCATTACGATAACCCGAAGCGTAAGAGATCGTATGTGAAGCCCGGCCAACCAGCCGAATCGAC 700

L H R I I T C D E K W I H Y D N P K R K R S Y V K P G Q P A E S T

ACCAAAGCCAAATATCCATGGCGCTAAGGTAATGCTCTGTATTTGGTGGGAGCAAAAGGGTCCTATCTATTATGAGCTGCTGAAATCTTTCCAGACCATC 800

P K P N I H G A K V M L C I W W E Q K G P I Y Y E L L K S F Q T I

ACAGGGAACCTGTACCGAACGCAACTGATTCGTTTGAAGCAAGCATTGGCCGAAAAACGCCCAGAATATGCGGCCAGACATGAAACCGTAATATTCCATC 900

T G N L Y R T Q L I R L K Q A L A E K R P E Y A A R H E T V I F H H

ATGACAACGCTCGGCCACATGTTGCAATACCTGTTAAAAACTATTTAGAAT/AGAAGTGGTTGGGAAGTTTTGCCTCACCCGCTTTATAGTCCAGACCTTGC 1000

D N A R P H V A I P V K N Y L E */R S G W E V L P H P L Y S P D L A

CCCGTCCGACTACTATTTGTTTCGATCGATGCAGAACGCTCTCTCTGGGATACGCTTCACTTTGGAACAGAGTATCCGAAATTGGCTTGATTCGTTCTTG 1100

P S D Y Y L F R S M Q N A L S G I R F T L E Q S I R N W L D S F L

GCCTCAAAAGATGAGCAGTTCTTTTGGCTCGGAATCCATATGTTGCCAGAAAGATGGGAAAAGGTCATAGCTAACACTGGCCAATACTTTGAATAAATTT 1200

A S K D E Q F F W L G I H M L P E R W E K V I A N T G Q Y F E *

ATATTGTACAAATGTTTCAAAATAAAAGCTAAAA/CATTAGACAGAATTCCGAATTTTTAAGTCATACACCCAATA 1275

TIR
