# Supplementary material for: Diversity of transposable elements and repeats in a 600 kb region of the fly Calliphora vicina
Source: Mob DNA. 2013 Apr 3;4:13. doi: 10.1186/1759-8753-4-13 (PMC3630058; doi:10.1186/1759-8753-4-13)
Supplement: Additional file 7: Figure S5 — ClustalW2 alignment of Cv-mar1 and Desmar1. [file 1759-8753-4-13-S7.doc]

Desmar1 TATTGGGTGTACAACTTAAAAACCGGAATTGGACGCTAGATGTCCACACTAACGAATAGT 60

Cv-mar1 ---------------TAAAAATGCGGGATTT-ACAATAGATGGCGTATCTTTTGAACACG 44

*:****: ***.*** **..****** * :.:**:: *** *

Desmar1 GTAAAAGCACAAATTTCATATATACGTCATTTTGAAGG-TACATTTGACAG-CTATCAAA 118

Cv-mar1 GTTTTTGTTTTTAAT-AACTTATATGTCATTTTGAAGGGTACATAT--CTGTCATTTATT 101

**::::* : ::*:* .* :**** ************* *****:* *:* *::* *::

Desmar1 ATCAGTCAATAAAACTATTCTATC-TGTGTGCATCATATTTTTTTATTAACTATGGAAAA 177

Cv-mar1 CTCACTTAGTTATTGAACATTATAGTGTAAACAACAAAGTTTTTTTTTG-CTTCG-AAAA 159

.*** * *.*:*:: :* : ***. ***.:.**:**:* ******:**. **: * ****

Desmar1 TTTTGAAAACTG-GCGAAAAAGACGGCATTTGCGGGAAGTTTTGTTGGGCCACTTTTTTG 236

Cv-mar1 TGTCGAATTTTGTGCCAACAAAGCGTCATATGCGGGAAGTTTTGCTTTACTTCTTTAATT 219

* * ***:: ** ** **.**..** ***:************** * .* :****::*

Desmar1 CAAAAAAAACTGCTGCAGAAAGTCACCGTTTGCTTGTGGAAGTTTACGGCGAACATGCCT 296

Cv-mar1 TGAAAAAAAGTGCCGCTGAAGCACACCGATTGCTCACCAAAGCTTATGGTGAATGTGTTT 279

.******* *** **:***. :*****:***** . .*** *** ** *** .** *

Desmar1 TAGCAAAAACACAGTGTTTCGAATGGTTTCAACGCTTCAAAAGTGGTGACTTTGATACGG 356

Cv-mar1 CATCGGTTTCAACGTGCGAGAGATGGTTTGTGCGGTTCAGAAGTGGTGATTTTGACACGG 339

* *..:::**..*** : ..******* :.** ****.********* ***** ****

Desmar1 AAGACAAAGAACGTCCTGGTCAGCCAAAAAAGTTTGAAGACGAAGAACTGGAGGCATTAC 416

Cv-mar1 AAGACAAAGATCGCCCAGGCCAGCCAAAAAAGTTTGAAGACCAAGAATTGGAGGCATTAC 399

**********:** **:** ********************* ***** ************

Desmar1 TCGATGAAGATTGTTGTCAAACGCAAGAAGAACTTGCAAAATCTTTAGGAGTGACACAAC 476

Cv-mar1 TCCATGAAGATTGTTGTAAAACTCAACAAGAGCTTGCAAAATCATTGGGAGCTACTCAAG 459

** **************.**** *** ****.***********:**.**** **:***

Desmar1 AAGCCATTTCAAAACGGCTAAAGGCAGCTGGATACATTCAAAAGCAAGGAAATTGGGTCC 536

Cv-mar1 CAGCAATTTCAAAACGTTTGCGAGCAGCAGGATTCATCCAAAAGCAGGGAAATTGGGTAC 519

.***.*********** *....*****:****:*** ********.***********.*

Desmar1 CACACGAATTGAAGCCGAGAGACGTTGAACGGCGATTTTGCATGTCGGAAATGCTTCTTC 596

Cv-mar1 CATACGAATTGAAGCCGAGAGACCTTGAAAGACGATTTTGCATGTCCGAAATGATGCTTG 579

** ******************** *****.*.************** ******.* ***

Desmar1 AACGCCACAAAAAGAAGTCATTTTTGAGTCGAATTATTACTGGAGATGAAAAATGGATTC 656

Cv-mar1 AACGCTATAAAAGAAAATCATTTTTGCACCGAATCATTACTTGCGATGAAAAATGGATCC 639

***** * ****..**.*********.. ***** ****** *.************** *

Desmar1 ACTACGATAATTCCAAGCGCAAAAAATCATATGTGAAGCGCGGCGGACGAGCCAAATCAA 716

Cv-mar1 ATTACGATAACCCGAAGCGTAAGAGATCGTATGTGAAGCCCGGCCAACCAGCCGAATCGA 699

* ******** * ***** **.*.***.********** **** .** ****.****.*

Desmar1 CACCAAAGTCGAATCTCCATGGCGCCAAGGTAATGCTCTGTATTTGGTGGGATCAGAGGG 776

Cv-mar1 CACCAAAGCCAAATATCCATGGCGCTAAGGTAATGCTCTGTATTTGGTGGGAGCAAAAGG 759

******** *.***.********** ************************** **.*.**

Desmar1 GTGTTTTGTATTATGAGCTATTGGAACCGGGTCAGACGATCACAGGGGACCTCTACCGAA 836

Cv-mar1 GTCCTATCTATTATGAGCTGCTGAAATCTTTCCAGACCATCACAGGGAACCTGTACCGAA 819

** *:* ***********. **.** * ***** *********.**** *******

Desmar1 CACAATTGATCCGTTTGAAGCAAGCATTGGCCGAAAAACGCCCGGAATATGCGAAAAGAC 896

Cv-mar1 CGCAACTGATTCGTTTGAAGCAAGCATTGGCCGAAAAACGCCCAGAATATGCGGCCAGAC 879

*.*** **** ********************************.*********...****

Desmar1 ACGGGGCGGTAATATTCCATCATGACAACGCTCGGCCACATGTTGCTTTACCGGTTAAGA 956

Cv-mar1 ATGAAACCGTAATATTCCATCATGACAACGCTCGGCCACATGTTGCAATACCTGTTAAAA 939

* *...* **************************************::**** *****.*

Desmar1 ACTATTTGGAAAACAGTGGATGGGAAGTTTTACCCCACCCGCCTTATAGCCCAGACCTTG 1016

Cv-mar1 ACTATTTAGAATGAAGTGGTTGGGAAGTTTTGCCTCACCCGCTTTATAGTCCAGACCTTG 999

*******.***:..*****:***********.** ******* ****** **********

Desmar1 CCCCTTCTGACTACCATTTGTTCCGGTCGATGCAGAATGACCTTGCGGGAAAACGCTTCA 1076

Cv-mar1 CCCCGTCCGACTACTATTTGTTTCGATCGATGCAGAACGCTCTCTCTGGGATACGCTTCA 1059

**** ** ****** ******* **.*********** *. ** * **.*:********

Desmar1 CTTCAGAGCAGGGTATCCGAAAATGGCTTGATTCATTCTTGGCCGCCAAGCCGGCGAAGT 1136

Cv-mar1 CTTTGGAACAGAGTATCCGAAATTGGCTTGATTCGTTCTTGGCCTCAAAAGATGAGCAGT 1119

*** .**.***.**********:***********.********* *.**. . *.*.***

Desmar1 TTTTTGAGAAGGGAATCCATGAATTGTCAGAAAGATGGGAAAAAGTCATAGCTTCAGATG 1196

Cv-mar1 TCTTTTGGCTCGGAATCCATATGTTGCCAGAAAGATGGGAAAAGGTCATAGCTAACACTG 1179

* *** .*.: *********.:.*** ****************.*********:....**

Desmar1 GGCAATACTTTGAATAATGCATTCATTCATTTTGTTATCGAAATAAAGCATTAATTTTCA 1256

Cv-mar1 GCCAATACTTTGAATAAATTTATATTGTACAAATGTTTCAAAATAAAAGCTAAAACATTA 1239

* ***************: ::*.:* * ::: *:**.*******. .*:**: :* *

Desmar1 CTAAAAAATTCCGGTTTTTAAGTTGTACACCCAATA 1292

Cv-mar1 GACAGAATTCCGAATTTTTAAGTCATACACCCAATA 1275

:.*.**:* * ..********* .***********
