# Supplementary material for: Diversity of transposable elements and repeats in a 600 kb region of the fly Calliphora vicina
Source: Mob DNA. 2013 Apr 3;4:13. doi: 10.1186/1759-8753-4-13 (PMC3630058; doi:10.1186/1759-8753-4-13)
Supplement: Additional file 8: Figure S6 — ClustalW2 alignment of Cv-mar1 and Desmar1 transposases. [file 1759-8753-4-13-S8.doc]

Cv-mar1_tp MSNFV--PTKRHMREVLLYFFNLKKSAAEAHRLLTKAYGECVSSVSTCERWFVRFRSGDF 58

Desmar1_tp MENFENWRKRRHLREVLLGHFFAKKTAAESHRLLVEVYGEHALAKTQCFEWFQRFKSGDF 60

*.** .:**:***** .* **:***:****.:.*** . : : * .** **:****

Cv-mar1_tp DTEDKDRPGQPKKFEDQELEALLHEDCCKTQQELAKSLGATQAAISKRLRAAGFIQKQGN 118

Desmar1_tp DTEDKERPGQPKKFEDEELEALLDEDCCQTQEELAKSLGVTQQAISKRLKAAGYIQKQGN 120

*****:**********:******.****:**:*******.** ******:***:******

Cv-mar1_tp WVPYELKPRDLERRFCMSEMMLERYKRKSFLHRIITCDEKWIHYDNPKRKRSYVKPGQPA 178

Desmar1_tp WVPHELKPRDVERRFCMSEMLLQRHKKKSFLSRIITGDEKWIHYDNSKRKKSYVKRGGRA 180

***:******:*********:*:*:*:**** **** *********.***:**** * *

Cv-mar1_tp ESTPKPNIHGAKVMLCIWWEQKGPIYYELLKSFQTITGNLYRTQLIRLKQALAEKRPEYA 238

Desmar1_tp KSTPKSNLHGAKVMLCIWWDQRGVLYYELLEPGQTITGDLYRTQLIRLKQALAEKRPEYA 240

:****.*:***********:*:* :*****:. *****:*********************

Cv-mar1_tp ARHETVIFHHDNARPHVAIPVKNYLERSGWEVLPHPLYSPDLAPSDYYLFRSMQNALSGI 298

Desmar1_tp KRHGAVIFHHDNARPHVALPVKNYLENSGWEVLPHPPYSPDLAPSDYHLFRSMQNDLAGK 300

** :*************:*******.********* **********:******* *:*

Cv-mar1_tp RFTLEQSIRNWLDSFLASKDEQFFWLGIHMLPERWEKVIANTGQYFE 345

Desmar1_tp RFTSEQGIRKWLDSFLAAKPAKFFEKGIHELSERWEKVIASDGQYFE 347

*** **.**:*******:* :** *** *.********. *****
