# Supplementary material for: Diversity of transposable elements and repeats in a 600 kb region of the fly Calliphora vicina
Source: Mob DNA. 2013 Apr 3;4:13. doi: 10.1186/1759-8753-4-13 (PMC3630058; doi:10.1186/1759-8753-4-13)
Supplement: Additional file 9: Figure S7 — Cv-mar2 consensus sequence. Consensus sequence of the Cv-mar2 element and amino acid sequence of its putative transposase. Underlined nucleotides correspond to the inferred TIRs (35 bp long); there are 5 nucleotide changes between the two TIRs of the consensus sequence. [file 1759-8753-4-13-S9.doc]

TACGAGGGTGCTTCAATAAGTnCGCGACTTTTTGAATTTCCCGGCTCTTAACTGAAAGGGCAACACTGCTCCTGTCAACAGGCATCTGTCAGTTGACTCC 100

TIR

TGTCAAAATTTTAACAAGCTGCGTCATTTAGTTTGTGTTTGACAGTCATTAATAGCAGACTACCTCGTGACTTGAGGAGAAATTGGAAAAAAGTGAATTT 200

L E K S E F

CGTCTGCTCATTAAGCATTATTTTTTGCAGAAAAAAACCATCACTCAAATAAAGGCTAAGCTTGATAAATACTATGGGAACTCTGCACCATCAATTTCAA 300

R L L I K H Y F L Q K K T I T Q I K A K L D K Y Y G N S A P S I S M

TGGTAAAAAAGTGGTTTACTGAATTTCGTTGTGGCCGTACAAGCACGGAAGATGCCGAACGTTCTGGACGCCCAGTTGAGGTCTCTACACCCGAAACAAT 400

V K K W F T E F R C G R T S T E D A E R S G R P V E V S T P E T I

TAAAAAAAATCACGATATGGTGTTGACCGATCGGAGATTGAAAGTGCGAGAGATTGTGGAAGCCATAGGCATCTCACATGGCTCAGTGGTTTCAATTTTG 500

K K N H D M V L T D R R L K V R E I V E A I G I S H G S V V S I L

AATGATTACTTGGGTATGAGAAAGCTTTCCGCAAGATGGGTGCCGCGTTTGCTCACAATTGACCAnAAACACAATCATGTGACAACTTCGTAGGAGTGTT 600

N D Y L G M R K L S A R W V P R L L T I D ? K H N H V T T S * E C L

TGGCGTTGTTCAACCGCAATATCGACGAGTTTTTGCGCCGTTTCGTAACCATGGACGAAACGTGAATCAACCTCAACACACCAGAGACCAAAGAACAGTC 700

A L F N R N I D E F L R R F V T M D E T * I N L N T P E T K E Q S

AAAACAGTGGGTTTCTCGGGGTGAATCGATGCCAAAGAAGGCCAAGGTGGATTTGTCAGCCAATAAAGTCATGGCGACTGTTTTTTGTTATGGACTCGGC 800

K Q W V S R G E S M P K K A K V D L S A N K V M A T V F C Y G L G

ATCATTCACATTGACTACCTTCAAAAGGGTAAAACACTCAATGGCGAATATTATTCAAACTTATTGGAGAGATTTAATGAAGATTTGAAGTAAAAACGAC 900

I I H I D Y L Q K G K T L N G E Y Y S N L L E R F N E D L K * K R Q

AGCATTTGGCCAAGAAAAAAATTCGTTTTCACCAGGACAATGTACGGGTGCACAAATGTGCAGTCTGCAAGGCAAAATTACATTAATTAGGCTACGAAAT 1000

H L A K K K I R F H Q D N V R V H K C A V C K A K L H * L G Y E M

GCTCTGTCTTCCATCCTATTCTCCGGATTTAGCCCCGAGTGACTATTTCTTGTTTCCAAACCTGAAGAAATGTCTCGACGGAAAGAGATTTGACTCCAAC 1100

L C L P S Y S P D L A P S D Y F L F P N L K K C L D G K R F D S N

GATGAAATCATCTCACAAACAAATACCTATTTTGATGACCTCGACAAATCCTATTTTTTGGAAGGGATAAAAAAATTGGAGAAACGTTGGACAAAGTGCA 1200

D E I I S Q T N T Y F D D L D K S Y F L E G I K K L E K R W T K C I

TAGAGCTCAAAGGAGACTATGTTGAAAAATAAAATAATTTTTTATCCAAAAACCTGTGTTTCATTCAAAAAGTCACGGACTTTTTGACCCGCCCTCGTA 1299

E L K G D Y V E K * TIR
