# Supplementary material for: Diversity of transposable elements and repeats in a 600 kb region of the fly Calliphora vicina
Source: Mob DNA. 2013 Apr 3;4:13. doi: 10.1186/1759-8753-4-13 (PMC3630058; doi:10.1186/1759-8753-4-13)
Supplement: Additional file 10: Figure S8 — ClustalW2 alignment of Cv-mar2 and Mariner1_DYa. [file 1759-8753-4-13-S10.doc]

Cv-mar2 TACGAGGGTGCTTCAATAAGTNCGCGACTTTTTGAATTTCCCG-GCTCTTAACTGAAAGG 59

Mariner1_DYa TACGAGGGTCGTTTGATAAGTCCGTGACTTTTTGTATTTGCCGCGCACCTG-CTCTAAAT 59

********* ** .****** ** *********:**** *** **:* *. ** :**.

Cv-mar2 GCAACACTGCTCCTGTCAACAGGCATCTGTCAGTTGACTCCTGTCAAAATTTTAACAAGC 119

Mariner1_DYa ACAACACTGCTCCTGTCAGCAGTTATCGATTAACAGCTGACTGTAAAATTTTGAGAAAGC 119

.*****************.*** *** .* *. :*. .****.***:*** *..****

Cv-mar2 TGCGTCATTTAGTTTGTGTTTGACAGTCATTAATAGCAGACTACCTCGTGACTTGAGGAG 179

Mariner1_DYa TGCGTTTTTTGGTTTGCGTTTTATAGGCATTGAAAGCAGACGATCTCGTGAATTTTAACG 179

***** :***.***** **** * ** ****.*:******* * *******.** :...*

Cv-mar2 AAATTGGAAAAAAGTGAATTTCGTCTGCTCATTAAGCATTATTTTTTGCAGAAAAAAACC 239

Mariner1_DYa AAAATGGAAAAAAGTGAATTTCGTGTGCTAATTAAGCATTATTTTTTGCGTAAAAAATCC 239

***:******************** ****.*******************. ******:**

Cv-mar2 ATCACTCAAATAAAGGCTAAGCTTGATAAATACTATGGGAACTCTGCACCATCAATTTCA 299

Mariner1_DYa ATCACCGAAACCAAGGAAAGACTTGATAAATATTATGGGGACTCTGCACCATCAATTTCA 299

***** *** .****.:*..*********** ******.********************

Cv-mar2 ATGGTAAAAAAGTGGTTTACTGAATTTCGTTGTGGCCGTACAAGCACGGAAGATGCCGAA 359

Mariner1_DYa ATGGTTAAGAAATGGTTTACTGAGTTCCGTTGTGGTCGTACCAGTACAAGTGATGCCGAA 359

*****:**.**.***********.** ******** *****.** **...:*********

Cv-mar2 CGTTCTGGACGCCCAGTTGAGGTCTCTACACCCGAAACAATTAAAAAAAATCACGATATG 419

Mariner1_DYa CGTTCAGGTCGCCCAAAAGAGGTCGTCATGCCAGAAATCGTCGACAAAATCCATGGAATG 419

*****:**:******.::****** * .**.**** ..* .*.****: ** *.:***

Cv-mar2 GTGTTGACCGATCGGAGATTGAAAGTGCGAGAGATTGTGGAAGCCATAGGCATCTCACAT 479

Mariner1_DYa ATATTGGATGATCGGAGAATGAAAGTGCGTGAGGTAGCTGAGGCTGTAGGCATCTCAACT 479

.*.***.. *********:**********:***.*:* **.** .***********..*

Cv-mar2 GGCTCAGTGGTTTCAATTTTGAATGATTACTTGGGTATGAGAAAGCTTTCCGCAAGATGG 539

Mariner1_DYa GAACGGGTACATCACATTTTACATGAATATTTGGACATGAAAAAGCTTTCCGCGCGATGG 539

*.. .**. :* ..*****..****:** ****. ****.************..*****

Cv-mar2 GTGCCGCGTTTGCTCACAATTGACCANAAACACAATCATGTGACAACTTCGTAGGAGTGT 599

Mariner1_DYa GTGCCGCGATTGCTCACACACGACCATAAGCGCAACCGTGTGACCATTTCAAAGGAGTGT 599

********:*********.: *****.**.*.*** *.******.* ***.:********

Cv-mar2 TTGGCGTTGTTCAACCGCAATATCGACGAGTTTTTGCGCCGTTTCGTAACCATGGACGAA 659

Mariner1_DYa TTGGCGATGTTCAACCGCAATCCAAACGAATTTTTGCGCCGTTTCGTTACCGTAGACGAA 659

******:**************. ..****.*****************:***.*.******

Cv-mar2 ACGTGAATCAACCTCAACACACCAGAGACCAAAGAACAGTCAAAACAGTGGGTTTCTCGG 719

Mariner1_DYa ACATGGATCCACCACACCACACCAGAGACCAAAGAACAATCAAGACAGTGGGTTTCTCCG 719

**.**.***.***:**.*********************.****.************** *

Cv-mar2 GGTGAATCGATGCCAAAGAAGGCCAAGGTGGATTTGTCAGCCAATAAAGTCATGGCGACT 779

Mariner1_DYa GGTGAACGTGCACCAAAGAAGGCCAAGGTGGGTCTGTCGGCCAACAAGGTCATGGCCACA 779

****** . .*******************.* ****.***** **.******** **:

Cv-mar2 GTTTTTTGTTATGGACTCGGCATCATTCACATTGACTACCTTCAAAAGGGTAAAACACTC 839

Mariner1_DYa GTTTTTTGGGATGCACAAGGTATCATTCACATCGATTACCTTGAAAAGGGTAAAACGATC 839

******** *** **:.** *********** ** ****** *************..**

Cv-mar2 AATGGCGAATATTATTCAAACTTATTGGAGAGATTTAATGAAGATTTGAAGTAAAAACGA 899

Mariner1_DYa ACCGGCGAATATTATTCAGAGCTTTTGGACAGATTCGATATTGATTTGAAGCAGAAACGA 899

*. ***************.* *:***** ***** .**.::********* *.******

Cv-mar2 CAGCATTTGGCCAAGAAAAAAATTCGTTTTCACCAGGACAATGTACGGGTGCACAAATGT 959

Mariner1_DYa CCGCATTTGGCGAAAAAAAAAGTGCTGTTCCATCAGGACAATGCACGGGTGCACACGTGT 959

*.********* **.******.* * ** ** ********** ***********..***

Cv-mar2 GCAGTCTGCAAGGCAAAATTACATTAATTAGGCTACGAAATGCTCTGTCTTCCATCCTAT 1019

Mariner1_DYa GTAGTCAGCATGGCAAAATTTCATAAATTGGGCTACGAACTGCTACCCCATCCAGCATAT 1019

* ****:***:*********:***:****.*********.****. *:**** *.***

Cv-mar2 TCTCCGGATTTAGCCCCGAGTGACTATTTCTTGTTTCCAAACCTGAAGAAATGTCTCGAC 1079

Mariner1_DYa TCTCCAGATTTAGCCCCCTGTGACTATTTTTTGTTTCCAAACATGAAGAAATGGCTCGGC 1079

*****.*********** :********** ************.********** ****.*

Cv-mar2 GGAAAGAGATTTGACTCCAACGATGAAATCATCTCACAAACAAATACCTATTTTGATGAC 1139

Mariner1_DYa GGTAAGAGATTCGGGTCAAATGAAGAGGTCATCACAGAAACAAACGACTATTTTGAGGGC 1139

**:******** *. **.** **:**..*****:** ******* ..********* *.*

Cv-mar2 CTCGACAAATCCTATTTTTTGGAAGGGATAAAAAAATTGGAGAAACGTTGGACAAAGTGC 1199

Mariner1_DYa CTTGAGAAAACCTATTATTTGGAAGGAATAAAAAAATTGGAAAAACGCTGGACTAAATGT 1199

** ** ***:******:*********.**************.***** *****:**.**

Cv-mar2 ATAGAGCTCAAAGGAGACTATGTTGAAAAATAAAATAATTTTTTATCCAAAAACCTGTGT 1259

Mariner1_DYa ATAGAGCTAAAAGGAGATTATGTTGAGAAATAAAACGCTTCTTTGACGAAAAAAATATAT 1259

********.******** ********.******** ..** ***.:* *****..*.*.*

Cv-mar2 TTCATTCAAAAAGTCACGGACTTTTTGACCCGCCCTCGTA 1299

Mariner1_DYa TTTATTCAAAAAGTCACGGACTTATCAAACGACCCTCGTA 1299

** ********************:* .*.* .********
