# Supplementary material for: Diversity of transposable elements and repeats in a 600 kb region of the fly Calliphora vicina
Source: Mob DNA. 2013 Apr 3;4:13. doi: 10.1186/1759-8753-4-13 (PMC3630058; doi:10.1186/1759-8753-4-13)
Supplement: Additional file 11: Figure S9 — ClustalW2 alignment of Cv-mar2 and Mariner1_DYa transposases. [file 1759-8753-4-13-S11.doc]

Cv-mar2_tp LEKSEFRLLIKHYFLQKKTITQIKAKLDKYYGNSAPSISMVKKWFTEFRCGRTSTEDAER 60

Mariner1_DYa_tp MEKSEFRVLIKHYFLRKKSITETKERLDKYYGDSAPSISMVKKWFTEFRCGRTSTSDAER 60

:******:*******:**:**: * :******:**********************.****

Cv-mar2_tp SGRPVEVSTPETIKKNHDMVLTDRRLKVREIVEAIGISHGSVVSILNDYLGMRKLSARWV 120

Mariner1_DYa_tp SGRPKEVVMPEIVDKIHGMILDDRRMKVREVAEAVGISTERVHHILHEYLDMKKLSARWV 120

**** ** ** :.* *.*:* ***:****:.**:*** * **::**.*:*******

Cv-mar2_tp PRLLTID?KHNHVTTS*ECLALFNRNIDEFLRRFVTMDET*INLNTPETKEQSKQWVSRG 177

Mariner1_DYa_tp PRLLTHDHKRNRVTISKECLAMFNRNPNEFLRRFVTVDETWIHHTTPETKEQSRQWVSPG 180

***** * *:*:** * ****:**** :********:*** *: .********:**** *

Cv-mar2_tp ESMPKKAKVDLSANKVMATVFCYGLGIIHIDYLQKGKTLNGEYYSNLLERFNEDLK*KRQ 236

Mariner1_DYa_tp ERAPKKAKVGLSANKVMATVFWDAQGIIHIDYLEKGKTITGEYYSELLDRFDIDLKQKRP 240

* ******.*********** . ********:****:.*****:**:**: *** **

Cv-mar2_tp HLAKKKIRFHQDNVRVHKCAVCKAKLH*LGYEMLCLPSYSPDLAPSDYFLFPNLKKCLDG 295

Mariner1_DYa_tp HLAKKKVLFHQDNARVHTCVVSMAKFHKLGYELLPHPAYSPDLAPCDYFLFPNMKKWLGG 300

******: *****.***.*.*. **:* ****:* *:*******.*******:** *.*

Cv-mar2_tp KRFDSNDEIISQTNTYFDDLDKSYFLEGIKKLEKRWTKCIELKGDYVEK 344

Mariner1_DYa_tp KRFGSNEEVITETNDYFEGLEKTYYLEGIKKLEKRWTKCIELKGDYVEK 349

***.**:*:*::** **:.*:*:*:************************
