# Supplementary material for: Diversity of transposable elements and repeats in a 600 kb region of the fly Calliphora vicina
Source: Mob DNA. 2013 Apr 3;4:13. doi: 10.1186/1759-8753-4-13 (PMC3630058; doi:10.1186/1759-8753-4-13)
Supplement: Additional file 12: Figure S10 — ITmDD37E_Cv1. Full nucleotide sequence of the DD37E element of C. vicina and aminoacid sequence of its transposase. Underlined nucleotides are the TIRs, and bold and underlined amino acids the catalytic domain. [file 1759-8753-4-13-S12.doc]

TAAGGAGTGAGATCGAAAAATTCTTAACACACGTTTTTCATTACATTTTTTAACCCAAGTATTATTTGAATTTTTTTTTGATCAAGTTACCTTTGAAAAA 100

TIR

CATGTCAGTTATTATGTGTAATGTCAATTATATTAATTTTTTTGTTAATCTGATTAAAATGAGTGACCAGAAAAAAGTGCGTACTGAAATTATTAAATAT 200

M S V I M C N V N Y I N F F V N L I K M S D Q K K V R T E I I K Y

TTTCAACAAAACCCAACTTGGTCTTACAAAAAGTTGGCCAAGCATACAAAGGTCTGCCGTCAAACTGTTTCCAATGTTATTAAACAGTACCGGGAGAACT 300

F Q Q N P T W S Y K K L A K H T K V C R Q T V S N V I K Q Y R E N L

TGTCAGTTGATAGAAAACCTGGTTCAGGTAGAAGGAATGGTCCACATGATGTTTCTAAAGCCAAAAAAATAGAACGCATTTTCAAAAGAGCTCCCAACAC 400

S V D R K P G S G R R N G P H D V S K A K K I E R I F K R A P N T

ATCCGGTAGGAAAGCAGCCCGGTTAGCTCAGTGCTCGGACTATTTGGTACGAAAAGTTAAAGCTAATGCAGGTTTAAAAACATACAAGGCTCAAAAAGTT 500

S G R K A A R L A Q C S D Y L V R K V K A N A G L K T Y K A Q K V

CCTGACAGGAACGCTACTAAAAATTTAGAGGCCAAAAACAGAGCACGGAAATTGAAGTCAAGTTTTATAAAAAAATATTCTTGCTGCATAATGGATGACG 600

P D R N A T K N L E A K N R A R K L K S S F I K K Y S C C I M D **D** E

AAACGTATGTTCTGGCAGATTTTTCGCAACTTCCAGGTCAAAAATTTTATGTTGCTGATGCTCGAGGGAATGTTGAAGAAAAGTTTAGGACCCAAAAGCA 700

T Y V L A D F S Q L P G Q K F Y V A D A R G N V E E K F R T Q K Q

GACAAAATTTCCCAGAAAGTTCTTGGTATGGCAAGCAATATGCAGTTGCGGCAAAAGAAGCCACTCATTTGTTACAACGGGCTCTATAAATACCGAAATT 800

T K F P R K F L V W Q A I C S C G K R S H S F V T T G S I N T E I

TACATCAAGGAATGTTTACAAAAAAGGCTGCTTCCATTCATAAGACTTCATAATGTGTCCACTTATTTTTGGCCTGACTTGGCATCCTGTCACTATGGCA 900

Y I K E C L Q K R L L P F I R L H N V S T Y F W P **D** L A S C **H** Y G K

AACAAGCCCTTGAGTGGTACAAGAACAATAATGTGGTATTTGTACCAAGAGAGGCAAATCCTCCAAACTGCCCGGAGCTAAGGCCAGTGGAGAGATATTG 1000

Q A L E W Y K N N N V V F V P R E A N P P N C P E L R P V **E**  R Y W

GGCTCTTGTTAAAAGAGAATTGAAGAGTACAAAAAAGGTGTCCAAAAGTGTGGTAGATTTTAAACGGAGATGGACTACATGTTCGAGCAAAGTGACAGAA 1100

A L V K R E L K S T K K V S K S V V D F K R R W T T C S S K V T E

AGCACTATAAAAACGTTAATGGAAGGGTTTCCGAAAAAGGTTCAAAATTTCATCACTAGTGATTAAAACTATAAAAATAATTTTTTTTGTAAATTGTAAT 1200

S T I K T L M E G F P K K V Q N F I T S D *

AATAATTTCAATCAAATAAAAAAAAAATTAAAGCTGTAAGTTTAGTGGTTTCTTTTTTATAAACATATATGTATGTTAAGAATTTTTCGATCTCACTCCTTA 1302

TIR
