# Supplementary material for: Diversity of transposable elements and repeats in a 600 kb region of the fly Calliphora vicina
Source: Mob DNA. 2013 Apr 3;4:13. doi: 10.1186/1759-8753-4-13 (PMC3630058; doi:10.1186/1759-8753-4-13)
Supplement: Additional file 13: Figure S11 — Helitron2_Cv consensus sequence. Consensus sequence of Helitron2_Cv showing the main structural features: 5′ and 3′ subTIRs and IR are underlined, 3′ stem loop in red and microsatellite repeat in blue. [file 1759-8753-4-13-S13.doc]

TT**ATACCCTACAC**CACCATAGTGGGG**AGGGTAT**TATGCGTTTGTGCAGATGTTTGTAACGCCCAAAAATATTAGTCTAACACCCACCTTAAAGTATACCG 100

**5’ subTIR IR**

ATCGACTTAGAATCACTTTCTGAGTCGATTAAACGAT**GTCCGTCCGTCCGTCTGGTCGGCTGGCTGGCTGTCC**ATGTAAACCTTGTGCGCAGAGTACAGG 200

Microsatellite repeat

TCGCAATTTTGAAGATATTTCGATGAAATTTGGTACATATTATTTTTTCGGCCCAAGGACCAAGCCTATTGAAACTGGCTGAAATCGGTCCATTATTTCA 300

CCTAGCCCCCATACAAATGTCCTCCCGAAATTGGACTTTATCGGTCATAAATGTTTAATTTATAAATGTATCTCCACAAATTGCGCTCCAAATAAGTTTT 400

ATATATACAAAATTCATGTCACCAAATTTTGTTACGATCGGTCCATAATTAGTCATAGCTCCCATATAGACCCGCTTCCGAAAATCACTTTAACGTGCAT 500

AAATCGCTTAAAAATGTTGGTATACACACAAAATTCAACATAGTAAACTTTCATATAGACATAAATCACACGACCTAATTTCATGGTGATCGGTCCATAA 600

TTGGTCATAGCCCCCATATAAGGCCCACTTCCGAAAATCACTCAAAAATATAAATTATTGAAATTTTAAAAGAAAAATGTTTTTACTCTTTTACTTA**GTG** 700

**TAGGGTAT**TA**TATGGTCGG**GCTTGA**CCGACCATA**CTTTCTTACTTGTTTT 750

**3’subTIR 3’ stem loop**
