# Supplementary material for: Diversity of transposable elements and repeats in a 600 kb region of the fly Calliphora vicina
Source: Mob DNA. 2013 Apr 3;4:13. doi: 10.1186/1759-8753-4-13 (PMC3630058; doi:10.1186/1759-8753-4-13)
Supplement: Additional file 14: Figure S12 — Helitron3_Cv consensus sequence. Alignment of the consensus sequence of the two subtypes of Helitron3_Cv (3a and 3b). The main structural features are highlighted: 5′ subTIR and IR underlined, 3′ stem loop in red and microsatellite repeat in blue. This element lacks the 3′subTIR. [file 1759-8753-4-13-S14.doc]

Helitron3a TTTT**TATACCCTTC**ACCTT-CGTGA**GAAGGGTATA**TATAAGTTTGTCATTCCGTTTGTAATTTCTACADTTTTCATTTCCGACCCTATAAAGTATATATA 99

Helitron3b TTAT**TATACCCTT**CACCATGAGTGGC**AAGGGTATA**TATAAGTTTGTCATTCCGTTTGTAATTTCTACATTTTTCATTTGCGACCCCACAAAGTATATATA 100

** ************** * *** ****************************************** ********* ****** * ************

**5’ subTIR IR**

Helitron3a TTCTGGATCCTTATAGATAGCGGAGTCGATTAAGCCAT**GTCCGTCTGTCT**GTTGAAATCAATTTTCTGAAGACCCCAGATATCTTCGGGATCCAAATCTT 199

Helitron3b TTCTGGATCGTTATAGATAGCGGAGTCGATATAGCCAT**GTCCGTCTGTCT**GTTGAAATCAACTTTCYGAAGCCCCCAAATAACTTACATACACGATTBAT 200

********* ******************** ********************************* **** **** ***** *** *** * * * *

**Microsatellite repeat**

Helitron3a CAATAATTCTGTCAGACATGCTTTCGAGAAKTTTGCTATTTAAAATCAGCAAAATCGGTCCACAAATGGCTGAGATATGAGGAAAAAACCARGACAACCT 299

Helitron3b ACATCAATATCTCCGAAATTCTTCCGGCTCGGTTGCTATTTAAAATCGAGAAAATCGGTCCACAAATGGCTGAGATATAAGGAAAAAACCAGGACAACCT 300

** * * * ** ** ** *** ** *************** **************************** ************ ********

Helitron3a CGATTTTTGACCTATTTTTGAYCTATATCTGGATTACTAARACATTAATATAGACAATATGG**ATATCTA**ATGA**TAGATAT**TTCAAAGACMTTTGCAA 395

Helitron3b CGATTTTTGACCTATTTTTKACCCATATCTGGATTACTAAGTCATTAATATAGACAATATGG**ATATCTA**ATGA**TAGATAT**TTCAAAGACCTTTGCAA 396

******************* * * **************** *********************************************** *******

**3’ stem loop**
