# Supplementary material for: Diversity of transposable elements and repeats in a 600 kb region of the fly Calliphora vicina
Source: Mob DNA. 2013 Apr 3;4:13. doi: 10.1186/1759-8753-4-13 (PMC3630058; doi:10.1186/1759-8753-4-13)
Supplement: Additional file 15: Figure S13 — Unknown5 consensus sequence. Consensus sequence of the Unknown 5 elements. The highly conserved region of the element is shown in red. [file 1759-8753-4-13-S15.doc]

AATATTTTTTAAACCGAAATTTTTTTTCATCAAAAATTTTTTTTTCCAAAAAAATTTAAAGAAATTTAAAAAAAAAATGTTTAAAAAACTTTATTTAAA 100

AATAATTAAAAAACAATTTCGAAAAAAAAATTTTTTAAAAAATTTTAAAACATTAAAAAAAATTTT**GATTTTGTTTAAATAAAAATATTTAAAAAAAAA** 200

**AATATTTTAAAGTATAATTTGGTGAAGGGTATATAAGATTCGGCACAGCCGAATATAGCTCTCTTACTTGTT** 275
