# Supplementary material for: Diversity of transposable elements and repeats in a 600 kb region of the fly Calliphora vicina
Source: Mob DNA. 2013 Apr 3;4:13. doi: 10.1186/1759-8753-4-13 (PMC3630058; doi:10.1186/1759-8753-4-13)
Supplement: Additional file 16: Figure S14 — Unknown6 consensus sequence. Consensus sequence of the Unknown 6 elements. The palindromic region is underlined. [file 1759-8753-4-13-S16.doc]

AAAATATAATTGGGCATGGTTACTGTAACCATTTAAATAGTGTTACAAGATTTTTAACAATATTATAGTCACAGTAACCATTTACATGATTGTGGTAAC 100

CATAATATGGTTAATTTACGATTCACATGATTGTATCAACCATATATATGGTTACAGTAAACAAATATATGTTTGTGGCAACCATATTTATGATGAATA 200

ATTTTATCATAnTATGTTGTTCTCAGATTATGATAAGCCTTAAGCCGAGAGCAACATAATATGATAAGTCCTTCATCATATAAATGGTTGTCACAAACA 300

TATATTTGTTTACTGTAACCATATATATGGTTGATACAATCATGTGAATCGTAAATTAACCATATTATGGTTACCACAATCATGTAAATGGTTACTGTG 400

ACTATAATATTGTTAAAAAACTTGTAACAATATTGAAATGGTTACAGTAACCATGCCCAACTATATTTTTTCTCTGCGTGTATATA 488
