# Supplementary material for: Diversity of transposable elements and repeats in a 600 kb region of the fly Calliphora vicina
Source: Mob DNA. 2013 Apr 3;4:13. doi: 10.1186/1759-8753-4-13 (PMC3630058; doi:10.1186/1759-8753-4-13)
Supplement: Additional file 17: Figure S15 — Unknown 20 consensus sequence. Consensus sequence of the unknown 20 elements. No structural features were identified. [file 1759-8753-4-13-S17.doc]

AGATTCTATATGTGTAATTTTTTTGAAATCGGAACACAAACGAAGAAATAGGATCGTTTTAAAAATGTAACATACCCGAGGTGTCCTACTTTGGGGACCC 100

CTGGTCGCGNCCCTGGTGGGCTTATCAGGTCCAAGTTCA 140
